# Supplementary material for: simAIRR: simulation of adaptive immune repertoires with realistic receptor sequence sharing for benchmarking of immune state prediction methods
Source: Gigascience. 2023 Oct 17;12:giad074. doi: 10.1093/gigascience/giad074 (PMC10580376; doi:10.1093/gigascience/giad074)
Supplement: giad074_GIGA-D-23-00048_Original_Submission [file giad074_giga-d-23-00048_original_submission.pdf]

GigaScience

simAIRR: simulation of adaptive immune repertoires with realistic receptor sequence sharing for benchmarking of immune state prediction methods

--Manuscript Draft--

**Manuscript Number:**	GIGA-D-23-00048
**Full Title:**	simAIRR: simulation of adaptive immune repertoires with realistic receptor sequence sharing for benchmarking of immune state prediction methods
**Article Type:**	Research
**Funding Information:**	Leona M. and Harry B. Helmsley Charitable Trust (2019PG-T1D011) — Dr Victor Greiff<br>UiO World-Leading Research Community — Dr Victor Greiff<br>UiO: LifeScience Convergence Environment Immunolingo — Dr Victor Greiff, Dr Geir Kjetil Sandve<br>EU Horizon 2020 iReceptorplus (825821) — Dr Victor Greiff<br>Norwegian Cancer Society Grant (215817) — Dr Victor Greiff<br>Research Council of Norway (300740) — Dr Victor Greiff<br>Research Council of Norway (331890) — Dr Victor Greiff<br>Research Council of Norway IKTPLUSS (311341) — Dr Victor Greiff, Dr Geir Kjetil Sandve<br>Stiftelsen Kristian Gerhard Jebsen — Dr Geir Kjetil Sandve
**Abstract:**	Background: Machine learning (ML) has seen an increase in interest in classifying immune states in adaptive immune receptor repertoires (AIRR) to aid the development of immunodiagnostics and therapeutics. Simulated data are crucial and necessary for the development and comprehensive evaluation of AIRR-ML methods e.g. through crowdsourced ML competitions. Results: We demonstrate that a common approach to generating simulated AIRR benchmark datasets can introduce biases, which may be exploited for undesired shortcut learning by certain ML methods. We devised a simulation strategy (simAIRR) to mitigate undesirable access to true signals in simulated AIRR datasets. simAIRR constructs antigen-experienced-like baseline repertoires by introducing signals that follow the empirical relationship between VDJ generation probability and population incidence of public sequences calibrated on real-world experimental datasets. By allowing users to provide a set of true immune state-associated sequences, simAIRR can be used for constructing repertoire-level benchmarks based on a range of assumptions (or experimental data source) for what constitutes receptor level immune signals. This includes the possibility of either making or not making any prior assumptions regarding the similarity or commonality of immune state-associated sequences that will be used as true signals. We demonstrate the real-world realism of our proposed simulation approach by showing that basic ML strategies perform similarly on simAIRR-generated and real-world experimental AIRR datasets. Conclusions: This study not only sheds light on the potential shortcut learning opportunities for ML methods that can arise with the state-of-the-art way of simulating AIRR datasets, but also provides a solution through a simulation strategy implemented as a Python package: https://github.com/KanduriC/simAIRR.
**Corresponding Author:**	Chakravarthi Kanduri, Ph.D<br>University of Oslo: Universitetet i Oslo<br>Oslo, NORWAY
**Corresponding Author Secondary Information:**	
**Corresponding Author's Institution:**	University of Oslo: Universitetet i Oslo

Powered by Editorial Manager® and ProduXion Manager® from Aries Systems Corporation

Corresponding Author's Secondary Institution:	
First Author:	Chakravarthi Kanduri, Ph.D
First Author Secondary Information:	
Order of Authors:	Chakravarthi Kanduri, Ph.D
	Lonneke Scheffer
	Milena Pavlović, PhD
	Knut Dagestad Rand, PhD
	Maria Chernigovskaya
	Oz Pirvandy
	Gur Yaari, PhD
	Victor Greiff, PhD
	Geir Kjetil Sandve, PhD
Order of Authors Secondary Information:	
Additional Information:	
Question	Response
Are you submitting this manuscript to a special series or article collection?	No
**Experimental design and statistics**<br><br>Full details of the experimental design and statistical methods used should be given in the Methods section, as detailed in our Minimum Standards Reporting Checklist. Information essential to interpreting the data presented should be made available in the figure legends.<br><br>Have you included all the information requested in your manuscript?	Yes
**Resources**<br><br>A description of all resources used, including antibodies, cell lines, animals and software tools, with enough information to allow them to be uniquely identified, should be included in the Methods section. Authors are strongly encouraged to cite Research Resource Identifiers (RRIDs) for antibodies, model organisms and tools, where possible.	Yes

Powered by Editorial Manager® and ProduXion Manager® from Aries Systems Corporation

Have you included the information requested as detailed in our Minimum Standards Reporting Checklist?	
**Availability of data and materials**<br><br>All datasets and code on which the conclusions of the paper rely must be either included in your submission or deposited in publicly available repositories (where available and ethically appropriate), referencing such data using a unique identifier in the references and in the "Availability of Data and Materials" section of your manuscript.<br><br>Have you have met the above requirement as detailed in our Minimum Standards Reporting Checklist?	Yes

Powered by Editorial Manager® and ProduXion Manager® from Aries Systems Corporation

Cover letter
Click here to access/download;Manuscript;cover_letter_simAIRR.pdf

Dear Editors,

We would like to submit our manuscript, entitled “simAIRR: simulation of adaptive immune repertoires with realistic receptor sequence sharing for benchmarking of immune state prediction methods”, as a research article in GigaScience. The manuscript fits well with the scope of the GigaScience journal both as a new algorithm/ computational method (under the technical note category) to simulate sequence data and also as a communication that brings awareness of potential pitfalls involved in simulations in this study field (under research article category). The software submitted with this article is central to the upcoming crowdsourced benchmarking competition in this field. For now, we prepared the manuscript suiting the "Research" article category.

There has been increasing interest in recognition of patterns embedded in DNA sequences of adaptive immune receptor sequence repertoires (AIRR) because of its promise in next-generation diagnostics and therapeutics. Machine learning (ML) is a key technology for distinguishing disease-associated rare sequence patterns from huge noise (one out of 10^5 sequences may contain immune signals) in AIRR-sequencing data. The application of ML to AIRR-seq data is growing at an exponential pace and a multitude of individual methods have been proposed: e.g., GLIPH (*Glanville, Nature, 2017*), TCRdist (*Dash, Nature, 2017*), DeepRC (*Widrich, NeurIPS, 2020*), DeepTCR (*Sidhom, Nat Comms, 2021*) and a statistical modelling approach (*Emerson, Nature Genetics, 2017*). To aid the widespread adoption of AIRR-ML, together with a large consortium of international collaborators, we recently developed a large domain-adapted ecosystem for ML on AIRR-seq data (*Pavlovic, Nature Machine Intelligence, 2021*). Last year, we also profiled the limits and capabilities of baseline AIRR-ML methods in an extensive benchmarking study published in GigaScience (*Kanduri, GigaScience, 2022*).

Evaluation and benchmarking of AIRR-ML methods have to rely largely on simulated data because of the limited experimental data with known ground truth. In this article, we not only demonstrate that a common way of simulating AIRR-sequencing datasets introduces biases that can lead to undesired shortcut learning opportunities by ML methods, but also provide a solution to this problem in the form of a novel simulation strategy and computational tool for the simulation of AIRR benchmark datasets. Notably, mitigating undesired shortcut learning opportunities for ML methods is currently being acknowledged as crucial in the machine learning field across study disciplines because of the risk of reduced generalizability of the methods and reproducibility crisis in general (*Geirhos 2020, Nature Machine Intelligence*). The simulation strategy that we devised is able to generate realistic benchmarking datasets for AIRR-ML methods (as demonstrated through large case studies) and is a very important and timely contribution in the context of future community-driven benchmarking competitions of AIRR-ML methods. We believe GigaScience to be a suitable venue for this manuscript as a full research article and we adhered to the standards of GigaScience by submitting a docker container, adhering to the DOME guidelines, and making all the data public permanently with a DOI. All the authors have approved the manuscript for submission and the content of the manuscript has not been published or submitted for publication elsewhere.

Sincerely,
Geir Kjetil Sandve

main manuscript file Click here to access/download;Manuscript;simAIRR.docx

simAIRR: simulation of adaptive immune repertoires with realistic receptor sequence sharing for benchmarking of immune state prediction methods

Chakravarthi Kanduri[1,2,#] (skanduri@uio.no), Lonneke Scheffer[1] (lonnekes@ifi.uio.no), Milena Pavlović[1,2] (milenpa@student.matnat.uio.no), Knut Dagestad Rand[1] (knutdr@math.uio.no), Maria Chernigovskaya[3] (mariia.chernigovskaya@medisin.uio.no), Oz Pirvandy[4] (pirvandy@gmail.com), Gur Yaari[4] (gur.yaari@biu.ac.il), Victor Greiff[3] (victor.greiff@medisin.uio.no), Geir K. Sandve[1,2,#] (geirksa@ifi.uio.no)

[1] Centre for Bioinformatics, Department of Informatics, University of Oslo, Norway
[2] UiORealArt Convergence Environment, University of Oslo, Norway
[3] Department of Immunology and Oslo University Hospital, University of Oslo, Norway
[4] Faculty of Engineering, Bar-Ilan University, Israel
[#]Correspondence

Abstract

Background: Machine learning (ML) has seen an increase in interest in classifying immune states in adaptive immune receptor repertoires (AIRR) to aid the development of immunodiagnostics and therapeutics. Simulated data are crucial and necessary for the development and comprehensive evaluation of AIRR-ML methods e.g. through crowdsourced ML competitions.

Results: We demonstrate that a common approach to generating simulated AIRR benchmark datasets can introduce biases, which may be exploited for undesired shortcut learning by certain ML methods. We devised a simulation strategy (simAIRR) to mitigate undesirable access to true signals in simulated AIRR datasets. simAIRR constructs antigen-experienced-like baseline repertoires by introducing signals that follow the empirical relationship between VDJ generation probability and population incidence of public sequences calibrated on real-world experimental datasets. By allowing users to provide a set of true immune state-associated sequences, simAIRR can be used for constructing repertoire-level benchmarks based on a range of assumptions (or experimental data source) for what constitutes receptor level immune signals. This includes the possibility of either making or not making any prior assumptions regarding the similarity or commonality of immune state-associated sequences that will be used as true signals. We demonstrate the real-world realism of our proposed simulation approach by showing that basic ML strategies perform similarly on simAIRR-generated and real-world experimental AIRR datasets.

Conclusions: This study not only sheds light on the potential shortcut learning opportunities for ML methods that can arise with the state-of-the-art way of simulating AIRR datasets, but also provides a solution through a simulation strategy implemented as a Python package: https://github.com/KanduriC/simAIRR.

Keywords: simulation of AIRR data, shortcut learning, benchmarking of machine learning methods, adaptive immune receptor repertoires, AIRR, ML

Background

High-throughput sequencing of adaptive immune receptor (AIR) repertoires (AIRRs) including B cell and T cell receptors (BCRs and TCRs) can provide a snapshot of the ongoing and past immune responses [1–7]. Decoding the information specific to various immune responses embedded in AIRRs has recently seen a surge in interest because of its potential in aiding the development of immunodiagnostics and therapeutics [1–7]. Recent studies have shown that sequences selected against a common antigen share similarities in sequence patterns and can be detected in multiple individuals

that experienced the antigen (so-called public responses) [6–12]. While convergent recombination and recombination biases are known to be the major contributors to public responses in general [7], the magnitude of public responses can vary depending on the species, cell types, cell subsets, chains and pairedness of sequences [13].

The pattern recognition capacity of machine learning (ML) methods has been increasingly utilized to learn the sequence patterns associated with immune states [5,14,15]. Many studies continue to develop and apply classical ML (supervised and unsupervised) and modern deep-learning methods to learn complex sequence patterns that can distinguish immune states [16–34]. A large majority of the methods mainly concentrate on the sequence patterns in the complementarity determining region 3 (CDR3) since the CDR3 loops of AIRs are known to be the key determinants of antigen specificity [29,35,36]. The continued rise in the development and application of AIRR-ML methods warrants rigorous benchmarking to compare the performance of methods. To aid the widespread adoption and performance evaluation of AIRR-ML methods, we recently developed immuneML, a platform for machine learning analysis of AIRR data [37]. By profiling the performance and limits of baseline ML methods across a wide range of ML challenges, we also recently highlighted the scenarios where novel AIRR-ML method development may be needed [38]. A rigorous evaluation of AIRR-ML methods both during development and benchmarking requires suitable benchmark datasets with known true signals [39,40] (hereafter signal refers to sequence patterns in AIRR-seq data that distinguish immune states). However, there are currently only a few large-scale datasets of immune state-associated donor repertoires available, and even these are of limited size (<1000 donors) and offer limited knowledge of true signals (only donor-level annotation, with no true information at the individual receptor level) [18,41–43]. Therefore, simulated AIRR datasets with artificially introduced discriminative sequence patterns at the individual receptor level play a central role in the rigorous evaluation of AIRR-ML methods [39,44–48].

A principal observation regarding the failure of modern ML methods is that many are related to unintended "shortcut" strategies adopted by ML methods [49]. Shortcuts can be defined as decision rules that work well on selected benchmark datasets but fail to generalize to other real-world datasets [49]. In the context of AIRR-ML, the field will benefit not only by avoiding shortcut strategies in discriminative learning but also by generating synthetic AIRR datasets devoid of shortcut opportunities. Below we describe a notable shortcut opportunity in simulated AIRR datasets that arises as a result of the state-of-the-art simulation approaches but is absent in real-world experimental datasets.

The AIRRs of a study cohort with a common immune state can be categorized into two components: (a) private sequences, which are seen only in one individual of the cohort and (b) public sequences, which are observed in more than one individual in the cohort. Previous studies have suggested two main mechanisms that determine the inter-individual sharing of sequences: (a) convergent recombination, where owing to the biases of the stochastic V(D)J recombination models [50,51], the probability of generating certain sequences is high and thus such sequences are observed in multiple individuals [6,52–55]. (b) selection/bias in receptor usage, where identical or similar sequences are observed in numerous individuals that share a common immune state due to being selected against a common antigen [16,17,21,30,56,57]. The observed publicness of sequences in a cohort (how frequent they are) is known to depend on the sampled cohort size as well as sequencing depth [57,58].

The probability of generating a specific CDR3 sequence (often referred to as *generation probability* in AIR context) is the sum of the probabilities of all recombination events that can generate the specific sequence [59]. The generation probability distributions of private sequences and public sequences in

any sampled cohort differ considerably, where the population incidence of sequences increases monotonically with an increase in generation probability (**Figure 1.a**). By deriving an empirical relation between the population incidence and generation probability of sequences, it may be possible to identify sequences that are observed with unlikely high population incidence given their generation probability (**Figure 1.a**) [19,30]. Such sequences can potentially be immune state-associated and will be referred to as outlier sequences throughout this manuscript. Since antigen-experienced repertoires may carry several immune state-associated receptor sequences accumulated over time (specific to distinct antigens), mining of public sequences in antigen-experienced repertoire cohort may reveal several outlier sequences irrespective of any particular immune state (**Figure 1.b**). In a real-world setting, there is thus no trivial relation between the presence of outlier sequences in a repertoire and a given immune state of interest. However, constructing synthetic AIRR datasets by sampling from known V(D)J recombination models alone [44,46–48], will result in naive repertoires that have not experienced any immune events and thus do not carry multiple outlier sequences like antigen-experienced repertoires do (**Figure 1.b**). When a selected group of the simulated naive repertoires are enriched for sequence patterns to represent an immune state for prediction methods, the publicity versus generation probability relation can in itself make these repertoires stand out through being the only repertoires in the simulated dataset containing outlier sequences, and thus provide shortcut opportunities for ML methods. By learning to directly connect the presence of outlier sequences to a particular immune state, a predictive method could perform very well on a benchmark based on a prediction strategy that exploits simulation artifacts instead of learning immune state-associated signals that are relevant for real-word application. This issue, which is referred to as shortcut learning in the machine learning field, is known to lead to a lack of generalization and unintuitive failures of ML methods, and has been suggested to be one of the main barriers to robust, fair, trustworthy and deployable machine learning [49]. For the sake of convenience, we hereafter refer to the bias of discordance between introduced signal and baseline repertoires in simulated AIRR data as "***generation probability discordance bias***".

In this study, we investigate the shortcut opportunity arising from generation probability discordance bias in simulated AIRR datasets and show that such shortcuts are absent in tested real-world experimental datasets. To mitigate the demonstrated shortcut opportunity in AIRR datasets, we present the simulation strategy, simAIRR. simAIRR provides a systematic approach for simulating AIRR datasets according to the assumptions that immune receptor binding is determined either by (i) the full CDR3 sequences, and is best addressed by learning appropriate similarity metrics for full CDR3s or by (ii) sub-sequence patterns such as k-mers. We also present case studies to demonstrate the utility of simAIRR, where simulated AIRR datasets are generated using both full-CDR3 assumption and sub-CDR3 motif assumption (k-mers) and subsequently used for evaluating suitable ML methods as a function of different witness rates and sample sizes. Here, witness rate refers to the rate at which signal occurs in the positive class *examples*. Note that the italicized term *examples* commonly used in ML literature refer to repertoires throughout this article.

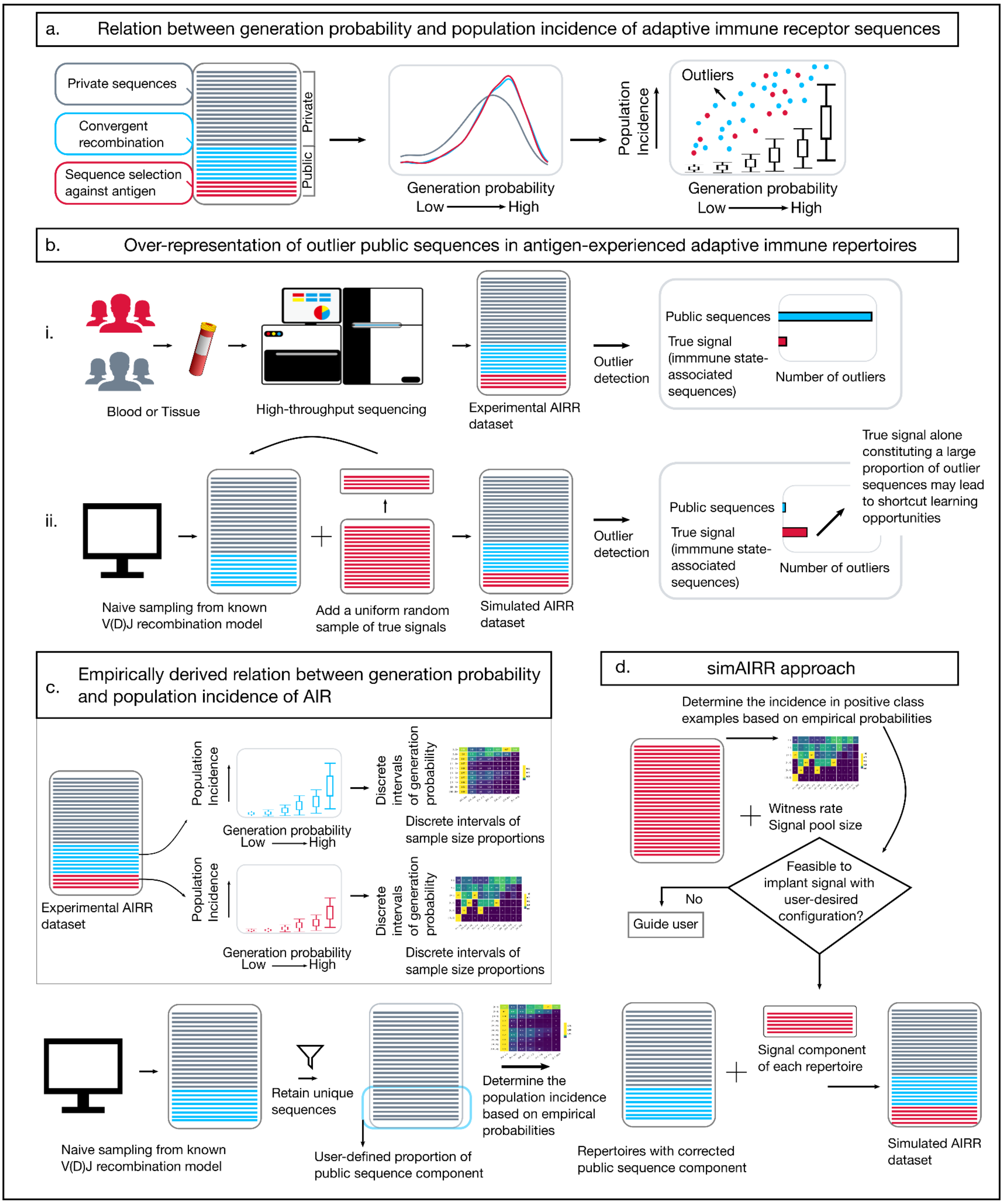

Figure 1: Rationale and simAIRR workflow. (a) Relation between generation probability and population incidence of adaptive immune receptor sequences: The generation probability distributions of private and public sequences (irrespective of being selected against a particular antigen) are known to differ. The relation between the generation probability and population incidence in a study cohort could be derived empirically. The empiric relation could be used to gauge outlier sequences that are observed with unlikely high population incidence given their generation probability. Note that the boxplots shown here are simplified for clarity and refer to the population incidence distributions within discrete intervals of generation probability of sequences. **(b) Over-representation of outlier public sequences in experienced adaptive immune repertoires:** (i) Real-world experimental AIRR datasets generated through high-throughput sequencing are expected to harbour many outlier public sequences because of accumulating multiple immune state-associated signals over time by being exposed to common antigens (with different signals being shared across different subsets of AIRRs). (ii) However, the same phenomenon is not naturally occurring in synthetic AIRR datasets generated by sampling from known V(D)J recombination models. When a single (label-associated) signal is introduced into synthetic repertoires as shown in ii, the added signal may stand out as outlier sequences unlike in experimental repertoires. This provides unintended shortcut opportunities for AIRR-

ML methods to detect the introduced signal. We refer to this shortly as "generation probability discordance bias". **(c) Empirically derived relation between generation probability and population incidence of AIR:** To mitigate unintended shortcut opportunities for AIRR-ML, we devised a novel simulation approach to generate AIRR datasets that rely on the empiric relation between generation probability and population incidence of public sequences calibrated from real-world experimental datasets separately for the presumed signal and other public sequences. For this, the user could either calibrate the relation on a real-world dataset of their choice or use the default choice we provide. **(d) simAIRR approach:** using the empirical relation between generation probability and population incidence of AIRs, simAIRR accepts a user-provided set of AIRs as a potential pool of immune state-associated signals and (i) determines whether it is feasible to introduce a signal into the baseline repertoires at the user-desired witness rate. If deemed feasible, simAIRR continues to (ii) generate baseline repertoires and (iii) adjust the proportion of public sequence components and generate repertoires with a corrected public sequence component, where the relation between generation probability and population incidence is respected. Note that the adjustment of the proportion of public sequence components is needed because, in datasets generated with naive sampling, there is low sequence sharing between repertoires, unlike experimental datasets. (iv) introduces signal components into the desired number of repertoires, where the relation between generation probability and population incidence is respected. If the signal introduction was deemed infeasible in (i), simAIRR provides descriptive information to the user to act as guidance in re-configuring the simulation. simAIRR could be used to execute the whole workflow (i, ii, iii, iv) in a sequence or exclusively to perform (i) or (ii) or (iii).

Analyses

Generation probability discordance bias in simulated AIRR datasets leads to unintended shortcut opportunities for ML methods

Throughout the manuscript, we refer to AIRs that are observed with unlikely high population incidence in a study cohort given their generation probability as outlier sequences. As an example of unlikely high population incidence given generation probability, consider a case where an AIR has a very low probability of occurring in > 1% of a population sample, but is rather observed in 10% of the population sample. We refer to the disparity in generation probability distributions between true signals and remaining public sequences in synthetic AIRR datasets as generation probability discordance bias. Here, true signals refer to the sequences that from the outset are known to be immune state-associated. In simulated datasets, true signals are those sequences introduced into the repertoires that distinguish the immune states. In the real-world experimental dataset used in this study, we refer to the original study-reported sequences as the true signals, for which the rationale is provided further below.

We hypothesised that, unlike real-world experimental datasets of antigen-experienced repertoires, simulated AIRR datasets may not carry several outlier sequences. Thus, the subsequent introduction of true signals representing an immune state will lead to shortcut opportunities that ML methods can exploit. To investigate whether generation probability discordance bias exists in real-world experimental and simulated AIRR datasets, we computed an outlier score for each public sequence of the experimental and simulated AIRR datasets irrespective of the immune state label (see Methods). The outlier score that we computed is qualitatively similar to the methodology by Pogorelyy et al. [19]. In addition to the outlier score, we computed a likelihood ratio for each public sequence (see Methods) that compares the probability of incidence of a sequence in contrasting immune states (positive and negative class labels in ML terminology). For this analysis, we specifically used three different datasets: (a) a real-world experimental T cell repertoire dataset with known cytomegalovirus (CMV) serostatus [18], (b) a human TCRβ sequence dataset simulated using a naive simulation approach. Here, naive simulation refers to sampling sequences from known V(D)J recombination models to construct synthetic repertoires and subsequent introduction of signals in a fraction of the repertoires to represent contrasting immune states. See **Figure 1.b** for a depiction. (c) a human TCRβ sequence dataset simulated using the simulation approach that we developed, simAIRR.

In simulated datasets, we know, by construction, the true signals that differ between immune states. In real-world experimental data, we are aware that experimental artefacts and other study design aspects could impact the selection of immune state-associated signals thus affecting what can be perceived as true signals. However, we considered the list of signals reported in the original study [18] as true signals for this analysis, as the purpose of this analysis is only to obtain an indication of the disparity between true signals and the remaining public sequences in terms of the degree of being outliers.

In the real-world experimental dataset [18], a decision rule based on thresholding the outlier measure that we computed (e.g. outlier measure >35) is observed to have very low precision (0.16%) and 78% recall (**Figure 2. a**) in retrieving the true signals. This indicates that the outlier measure alone is a poor classifier of the perceived true signals in real-world experimental data. On the contrary, in a naive-simulated AIRR dataset of TCRs, the same outlier measure-based decision rule is found to have 99% precision and 90% recall (**Figure 2. b**) in retrieving the true signals. This indicates that the outlier measure alone can capture a large fraction of the true signals in naive-simulated data, which can be used for shortcut learning by ML methods. To overcome the generation probability discordance bias demonstrated in **Figure 2. b**, we devised a novel simulation approach to construct benchmark AIRR datasets for the immune state prediction problem. In AIRR TCR datasets simulated based on our novel simulation approach, the same outlier measure-based decision rule was found to have 0.09% precision and 83% recall (**Figure 2. c**). Overall the very low precision equivalent to that observed in a real-world experimental dataset mitigates the shortcut opportunities that can arise through naive simulation approaches.

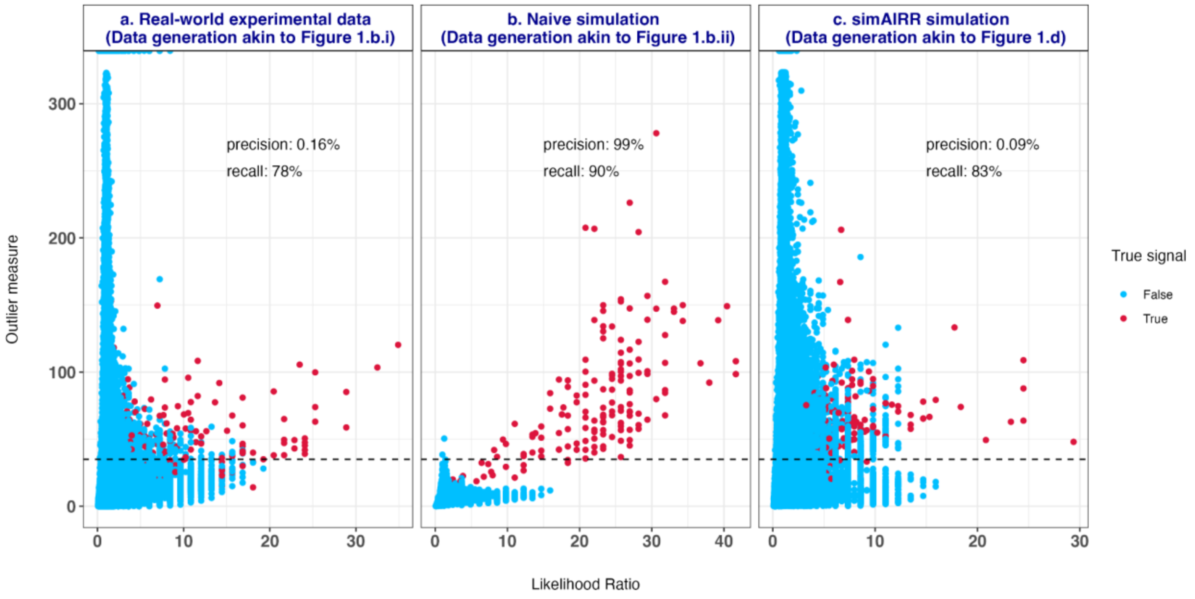

Figure 2: Generation probability discordance bias in the naive simulation of AIRR datasets. (a). In a real-world experimental AIRR dataset (n=683 repertoires; 307 positive class examples and 376 negative class examples, average unique number of TCRβs per repertoire ~ 200k), the outlier measure (y-axis) alone is a poor classifier of presumed true signals as reported in the original study. Here, each point represents a unique TCRβ CDR3 sequence and the colours represent whether the sequence is a true signal e. Red points are the true signal sequences. (b) On the contrary, in a synthetic dataset generated through a naive but intuitive simulation approach as shown in Figure 1.b.ii (n=683 repertoires; 310 positive class and 373 negative class examples), the outlier measure alone has a very high precision (99%). This behaviour of the simulation approach can lead to shortcut learning opportunities for ML methods. (c). Our novel simulation approach that is intended to mitigate the shortcut learning opportunity makes the repertoires antigen-experienced-like to behave more like the real-world experimental data, where there can be many outlier sequences because of accumulating many immune state-associated sequences over the lifetime of repertoires. On a dataset with matched sample size (n=683 repertoires; 310 positive class and 373 negative class

examples), our simulation approach reduces shortcut learning opportunities because thresholding on the outlier measure alone has a precision of 0.09%, which is comparable to the 0.16% precision of the real-world experimental data.

A novel simulation approach to mitigate shortcut opportunities for AIRR-ML methods

To mitigate unintended shortcut opportunities for AIRR-ML, we devised a novel simulation approach to generate AIRR datasets that rely on the empiric relation between generation probability and population incidence of public sequences calibrated from real-world experimental datasets separately for signal and other public sequences (**Figure 1. c**). We hereafter refer to this simulation approach as simAIRR. Below we briefly describe the simAIRR approach (see also **Figure 1.d**).

simAIRR approach: To determine the population incidence of public sequences (including both signal sequences and other public sequences), simAIRR relies on the empirical relation between generation probability and population incidence of AIRs. For this, the user could either calibrate the aforementioned relation based on a real-world dataset of their choice and supply the learnt relation to simAIRR, or use the default choice that is supplied with simAIRR. In simAIRR, the default models for the empirical relation between generation probability and population incidence of AIRs are based on a previously published large cohort study of TCR repertoires [18] calibrated separately for signal sequences (detected in the original study) and other public sequences. See the Methods section for details on deriving the empirical relation between generation probability and population incidence of AIR sequences. **Figure 3** shows the empirical distribution models learnt for the signal and other public sequences from the dataset of Emerson et al. [18]. The heatmaps show that a large fraction of the public sequences are present in a small fraction of the sampled population across all the generation probability distribution bins except for the high generation probability bins [8, 2) and [9, 8), where sequences with high generation probability are unsurprisingly observed in higher fractions of the sampled population. The empirical probability distributions differed between the presumed signal sequences and remaining public sequences, where the presumed signal sequences (full AIRs) occurred in a relatively higher fraction of the sampled population, unlike a large majority of the public sequences.

Using the empirical relation between generation probability and population incidence of AIRs, simAIRR accepts a user-supplied set of AIRs as a potential pool of immune state-associated signals and (i) determines whether it is feasible to introduce the signal sequences into the baseline repertoires at the user-desired witness rate (**Figure 1.d**). If deemed feasible, simAIRR continues to (ii) generate baseline repertoires and (iii) adjust the proportion of public sequences (the sequences that will be shared across repertoires in a dataset) and their population incidence levels. The public component correction is needed because datasets generated through naive sampling lack sequence sharing between repertoires to the degree that is observed in experimental datasets containing antigen-experienced repertoires (see Methods for details). In the repertoires generated with the corrected public sequence component, the relation between generation probability and population incidence is respected. (iv) simAIRR further introduces signal components into the desired number of repertoires, where again the relation between generation probability and population incidence is respected. If the signal introduction was deemed infeasible in (i), simAIRR provides descriptive information to the user to act as guidance in re-configuring the simulation. simAIRR could be used to execute the whole workflow (i, ii, iii, iv) in a sequence or exclusively to perform (i) or (ii) or (iii).

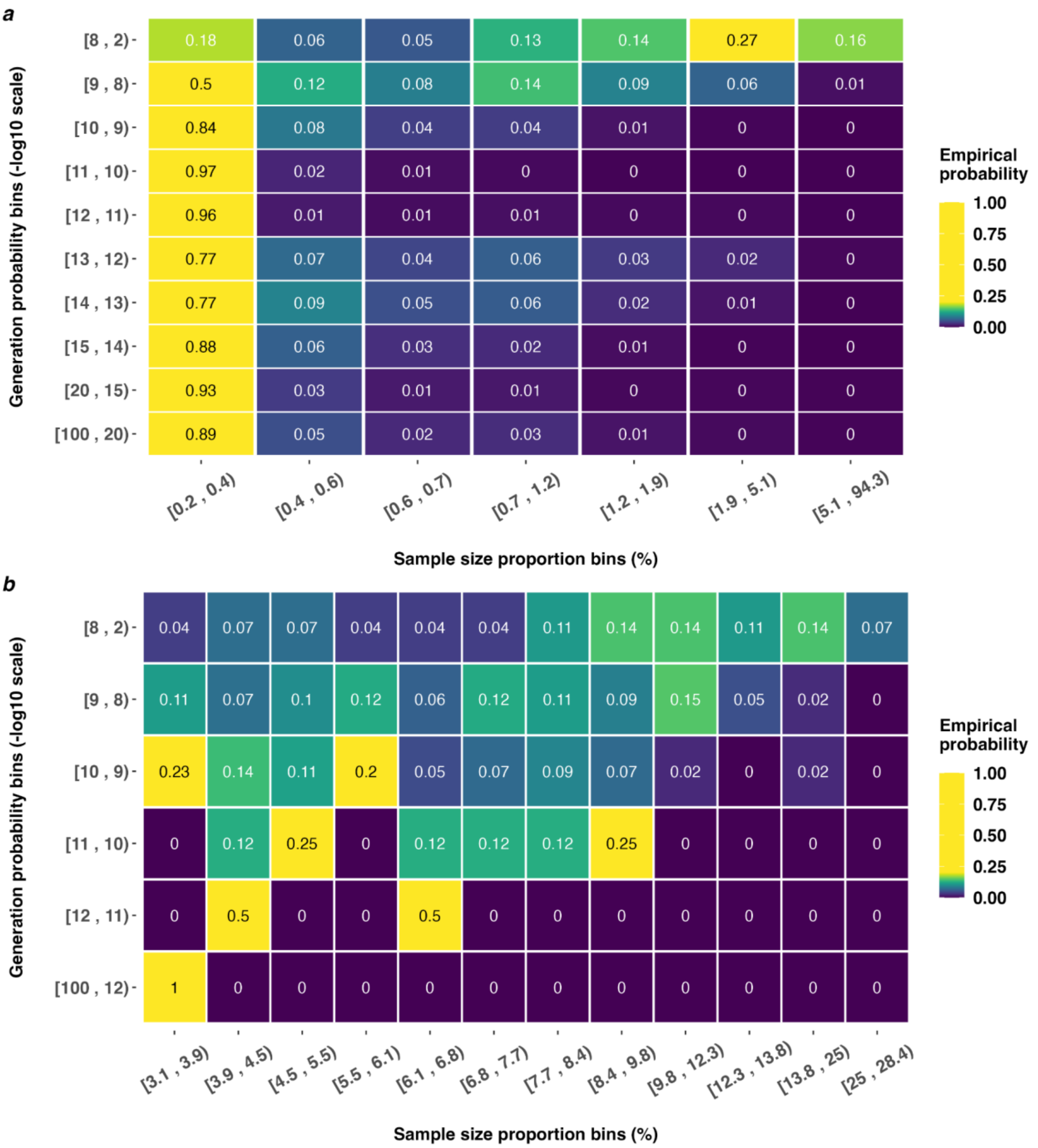

Figure 3: Empirical relation between generation probability and population incidence of public AIR sequences. The relation between generation probability and population incidence of public AIR sequences was determined based on a previously published large cohort study of TCR repertoires [18] separately for signal (**3.b**) and all the remaining public sequences (**3.a**). In both **a** and **b,** the x-axis represents the sample size proportion bins and the y-axis represents generation probability distribution bins in the -log10 scale. Note that the bins on both axes are half-open intervals that include the left endpoint but exclude the right endpoint. To explain one cell of the heatmaps, consider the top-left cell in panel **a**. The cell corresponding to the generation probability bin [8, 2) and sample size proportion bin [0.2, 0.4) tell that 18% of the total unique public sequences with a generation probability between [8, 2) (in -log10 scale) occur between [0.2, 0.4) % of total repertoires in the sample (dataset). The row-sums of the heatmaps should sum to 1.

Case studies

To demonstrate how simAIRR may be used to generate benchmark datasets for AIRR-ML predictions, we performed two separate case studies.

Case study 1: Prior assumptions on the similarity of immune state-associated sequences.

In the first case study, we made prior assumptions on how the immune state-associated sequences that differentiate the positive and negative class labels are similar. Specifically, we assumed that immune state-associated sequences share sequence similarity in the form of shared contiguous sub-sequences of size 4 (4-mers)

First, we generated independent AIRR datasets each containing 200 repertoires using simAIRR, where the average sequence count was 119,633 (± 1,313 s.d.). The datasets were simulated in such a way that 100 repertoires carried condition-associated sequences, hence labelled as positive class, whereas the negative-labelled repertoires did not receive any condition-associated sequences although they were not checked for carrying those specific sequences just by chance. We varied the average witness rate in different experiments to observe how the performance of the tested ML method varies depending on the average witness rate. We assumed that the signal sequences carry any one of the three 4-mers: *WKDY, YREV, and ERFY*. For this, instead of the implantation of k-mers as in our previous study [38], we selected sequences enriched for these 4-mers by querying a large set of reference sequences for matching patterns. To make such a pattern-matching process easy for users if need be, we provide a simple python script and a corresponding tutorial that shows how to generate a large set of reference sequences and retrieve the pattern-matched sequences of interest with minimal effort and with very few lines of code specification [60]. Notably, we here did not exclude the low probability events of a signal sequence carrying two or all the three k-mers of interest by chance, but the users can impose such additional sanity checks if need be. The rationale behind the choice of the selected k-mers was to compare the performance of ML methods on benchmark datasets with similar characteristics (witness rates and sample sizes) in our previous study that used the same k-mers. The signal sequences were added to the positive-class repertoires using the simAIRR approach, where the observed frequency of signal sequences in the dataset depends on the generation probability distributions calibrated based on real-world experimental data. Since the signal sequences are assumed to share any of the three chosen 4-mers, we used an ML method that matches the assumption that the signal is in the form of 4-mers as in our previous study [38]. Specifically, we used a highly-regularized logistic regression model that is well-optimized for hyperparameters on a 4-mer encoded representation of the amino acid sequences. To compare with the reported performance metrics of Emerson et al., [18], immuneML case studies [37], and Motifbooster [61], we here chose to present ROC AUC obtained through nested cross-validation. As expected, we observed that the performance of the suited ML method was close to perfect when the witness rate was equal to or above 10 sequences per 10^5 sequences (**Figure 4.A**). When the witness rate was 5 sequences per 10^5 sequences, we observed a ROC AUC of around 0.8 on average (**Figure 4.A**) and these observations were in strong alignment with what we previously observed on similar assumptions of signal sequences at similar witness rates [38].

Case study 2: No prior assumptions on the immune state-associated sequences.

In the second case study, we did not make any prior assumptions regarding the immune state-associated sequences, specifically in what way they are similar. We retrieved sequences that were reported to be specific to the Hepatitis C virus (HCV) from VDJdb [56] thereby avoiding making any prior assumptions regarding the similarity of signal sequences. We used a uniform random sample of the

HCV-associated sequences as the pool of signal sequences introduced into the positive-labelled repertoires.

Unlike in the first case study, we fixed the witness rate as constant in all the experiments (to have on average 10 immune state-associated sequences per approximately 10^5 sequences). Rather, we varied the sample size (number of examples) of the class-balanced AIRR datasets (200, 400 or 600 examples per dataset) to observe how the performance of a suited ML method varies depending on the sample size. The class balance and signal introduction were similar to case study 1, where 50% of the repertoires carried signal sequences. Since we do not have prior knowledge of the sequence similarity patterns of the signal sequences, we used a suited ML method that assumes the full sequence identity as a potential signal representation [18]. Specifically, we used a probabilistic binary classifier based on phenotype burden [18] implemented in immuneML [37], where the hyperparameters were selected through nested cross-validation. that is optimized well for the best hyperparameters. To compare with the reported performance metrics of Emerson et al., [18], immuneML case studies [37], and Motifbooster [61], we here chose to present ROC AUC obtained through nested cross-validation. The findings of the performance metrics align well with the known behaviour of the probabilistic binary classifier method [18], where prior studies reported a drop in the performance of the method at lower sample sizes [37,61]. When the sample size reached a similar level as in Emerson et al. [18] (n=600)**,** the ROC AUC reached a comparable level as reported in the original study [18] and other studies that re-analysed the same dataset [37,61] (**Figure 4.B**).

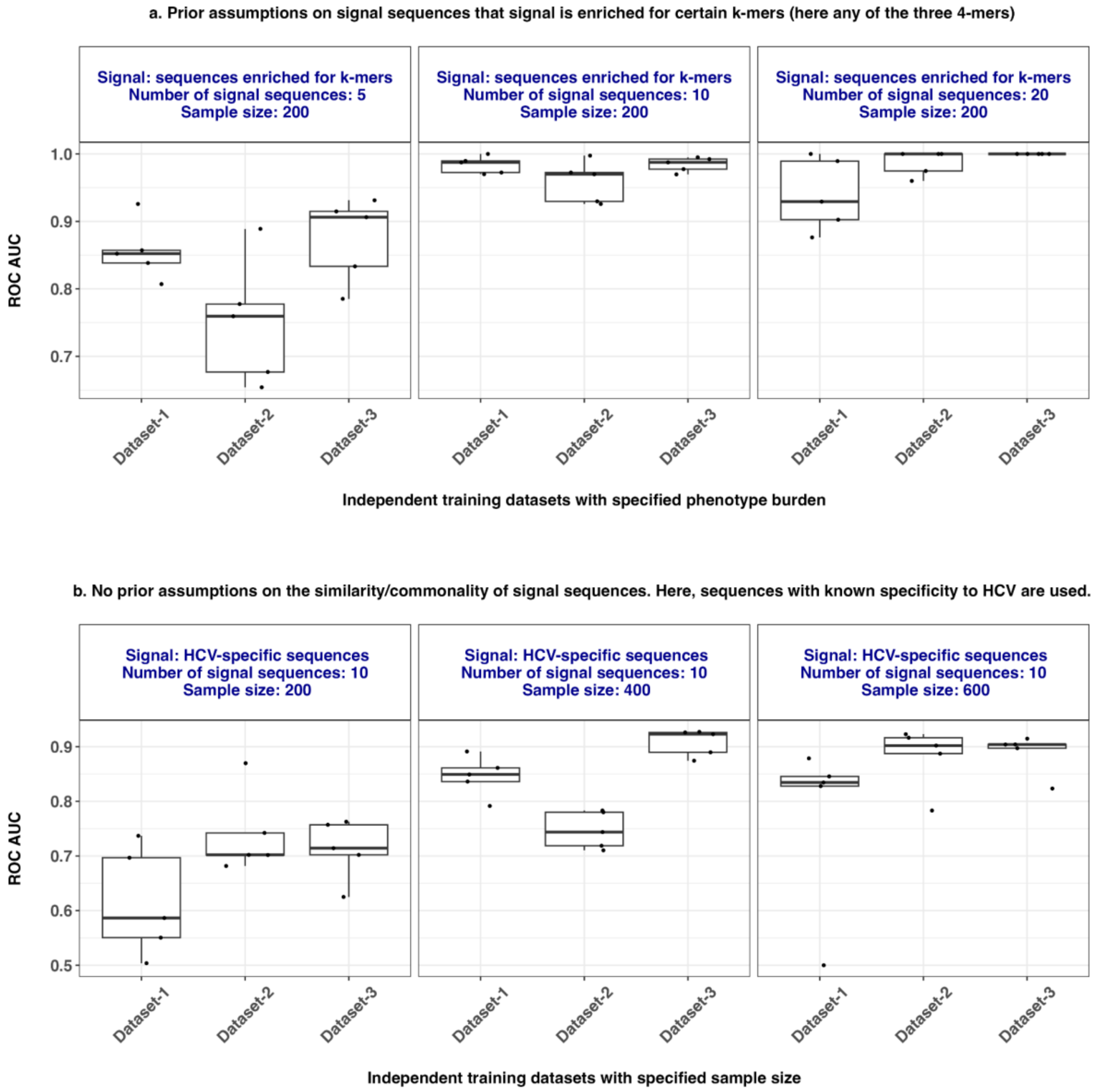

Figure 4: Performance of ML methods on benchmark datasets generated by simAIRR. **(A)** Performance estimates (ROC AUC) of a highly-penalized logistic regression model (on the y-axis) that is optimized well for hyperparameters in a binary classification of balanced, labelled AIRR datasets (number of examples=200) encoded as 4-mers, where the signal in positive class examples is composed of full sequences that share any of the three 4-mers: *WKDY*, *YREV*, *ERFY*. The rationale behind the choice of the selected k-mers was to compare the performance of ML methods on benchmark datasets with similar characteristics (witness rates and sample sizes) in our previous study that used the same k-mers. The full sequences sharing any of the three chosen 4-mers were obtained by generating a large number of sequences and retrieving only those sequences that carry the 4-mers through pattern matching. The sub-panels represent witness rates, where we vary whether the positive-labelled repertoires carry either 5, 10 or 20 immune-state-associated sequences per approximately 10^5 sequences. In each sub-panel, we simulated three independent datasets (named Dataset-1, Dataset-2 and Dataset-3) to gauge the variation in performance on similar dataset characteristics. The spread of the performance metrics on the y-axis shows the variation of performance obtained through nested cross-validation. (**B**) Performance estimates (ROC AUC) of a probabilistic binary classifier [18] implemented in immuneML [37] (on the y-axis) in a binary classification of balanced, labelled AIRR datasets (constant witness rate of 10 immune-state-associated sequences per approximately 10^5 sequences). The signal in positive class examples is composed of full sequences that are reported to have specificity for HCV. The HCV-specific sequences were downloaded from VDJdb [56]. The sub-panels represent datasets of different sample sizes of balanced AIRR datasets. In each sub-panel, we simulated three independent datasets (named Dataset-1, Dataset-2 and Dataset-3) to gauge the variation in performance on similar dataset characteristics. The spread of the performance metrics on the y-axis shows the variation of performance obtained through nested cross-validation.

Discussion

One major challenge in using simulated datasets for benchmarking machine learning (ML) methods is to prevent shortcut learning opportunities [62]. Shortcut learning may lead to biased benchmarking of AIRR-ML methods, reduced generalizability of the methods and reproducibility crisis [63]. Our study shows that an intuitive and state-of-the-art approach to generating simulated AIRR benchmark datasets can introduce signal artefacts that can be exploited for undesired shortcut learning by AIRR-ML methods. We refer to the introduced signal artefact as the generation probability discordance bias, where a large disparity exists in the generation probability distributions between the introduced true signals and the remaining public sequences. This can allow ML methods to exploit this bias and learn shortcuts instead of the true sequence patterns associated with immune states in the data. An analogy of such shortcut learning from the image recognition domain is an ML model trained to detect different types of animals in pictures (like cats and dogs) identifying the animals based on the background colour of the images rather than learning the patterns associated with animal objects.

To mitigate this problem, we developed simAIRR, a novel simulation strategy that constructs antigen-experienced-like baseline repertoires in the sense of publicity-generation probability relation. It introduces signals in the repertoires by following the empirical relationship between generation probability and sharing patterns of public sequences calibrated from real-world experimental datasets. This approach ensures that the simulated datasets are not biased and are instead representative of real-world scenarios. Our findings suggest that this novel simulation approach effectively mitigates the shortcut opportunities that can arise through naive simulation approaches. Notably, there exists no other tool/algorithm to achieve the goals of simAIRR as formulated in this study to make comparisons.

One key benefit of simAIRR's approach is the possibility of not making any prior assumptions regarding the similarity or commonality of immune state-associated sequences that will be used as true signals. Using previously known antigen-specific sequences from public databases (e.g., VDJdb [56,64,65]) or other experimentally-determined antigen-specific sequences as the pool of signal sequences, users can avoid making any prior assumptions regarding the similarity or commonality of signal sequences. This will be useful in representing real-world scenarios well because there is currently very limited knowledge of sequence similarity patterns between sequences selected against a common antigen. Existing AIRR-ML methods make varying assumptions regarding the similarity patterns of sequences selected against a common antigen. While some methods assume that signal sequences share sub-sequence patterns (k-mers) [20,22,29,38,66–68], other studies assume that immune receptor binding is determined by the full CDR3 sequence, and is best approached by learning appropriate similarity metrics for full CDR3s [18,41,69]. Using case studies, we demonstrated the utility of simAIRR in generating benchmark datasets for immune state prediction problems following varying assumptions regarding true signals: i.e., it could be assumed that the true signal sequences share sub-sequence patterns (k-mers, gapped k-mers, hamming distance etc) or known antigen-associated sequence pool could be used with no *a priori* assumptions.

The cases presented in this study demonstrate that simAIRR can generate AIRR benchmarking datasets for immune state prediction problems that represent real-world scenarios well. The behaviour of ML methods on the generated benchmarking datasets matches the behaviour of the same methods on real-world experimental datasets at different sample sizes and witness rates. This suggests that the generated benchmarking datasets sufficiently match the real-world experimental datasets in terms of signal and noise. Often, benchmarking of computational methods published as part of articles presenting novel methods identify the proposed novel method(s) as a winner(s), largely owing to benchmarking not being

fully neutral [70,71]. However, neutral benchmarking is especially valuable for the scientific community to make the evaluation of the methods more rational and to establish standards on a scientific basis [70,71]. We encourage AIRR-ML researchers to evaluate the performance of novel ML methods on neutral benchmarking datasets developed by other researchers to improve the generalizability of methods. For instance, the benchmarking datasets used in the case studies and similar simulation parameters at smaller sample sizes and lower witness rates represent suited benchmarking datasets for unbiased performance evaluation of novel AIRR-ML methods. These scenarios also represent the cases where novel AIRR-ML method development is needed because the performance of state-of-the-art ML methods drops compared to easier scenarios. Notably, the empirical performance maps of baseline ML models across a wide range of study design and ML challenges as profiled in our previous study [38] needs to be re-evaluated with more realistic simulated datasets (such as the ones generated in this study) given the strong prior assumptions on what constitutes a signal.

An important aspect to keep in mind is that the default models supplied with simAIRR with respect to the relation between generation probability and the population incidence of public sequences are calibrated based on human TCRβ chain sequences. However, the magnitude of public responses can vary depending on the species, cell types, cell subsets, chains and pairedness of sequences [13]. Thus, when intending to simulate AIRR datasets of different species, cell types and chains, users need to calibrate the relation between generation probability and population incidence of sequences on corresponding experimental datasets and supply such custom models to simAIRR. The file formats for these custom models are described in simAIRR's documentation. Note that the population incidence of sequences can be computed with CompAIRR [27] and the generation probability of sequences can be computed using OLGA [47] with minimal effort.

A plethora of studies have compared repertoires of a common immune state and found identical or similar sequences detected in many individuals [6–11]. AIRR-ML methods rely on this observation that condition-associated sequences or similar sequences will be observed in a considerable fraction of the sample size that shares the immune state. This overlap of similar signal sequences across repertoires is a post-V(D)J recombination phenomenon. Although repertoire generation models of B cell repertoires have been suggested to be individual-specific [72] (moderately mediated by a high degree of polymorphisms in *IGHV* genes), condition-associated similar sequences can be shared among individuals of a common immune state [6–11]. simAIRR simulates this exact phenomenon of shared similar sequences across repertoires while not allowing the sharing pattern to exceed what can be possible given a rough indication of the generation probability of the sequences. Previous research has suggested a high degree of concordance in repertoire generation models across individuals [48,59,73,74], however, recent observations provided evidence that the generation probability of sequences, in some cases, can vary up to or more than three orders of magnitude due to individualized recombination models [72]. It remains to be investigated how the individualized recombination models affect the overlap of public sequences at a population level and subsequently the pattern recognition capacity of ML models. If deemed necessary, future improvements of simAIRR should use individualized recombination models.

An important consideration for AIRR data simulation for ML-based prediction problems in the context of individualized repertoire generation models is what is the exact consequence of individualized repertoire general models at a population level. Although there exists limited knowledge on this aspect, we are allowing ourselves to speculate on the possible consequences. As individualized repertoire generation models and the generation probability of sequences are tightly related, some sequences that can be thought to be generated with high probability, in general, might not be occurring in individual

repertoires with the same probability leading to less overlap of sequences between repertoires at a population level. In simAIRR, users can customise the degree of sharing of public sequences across repertoires, meaning that the proportion of unique sequences in a repertoire dataset that is public can be controlled. A suitable default for this customisable parameter can be estimated from experimental datasets as a post-VDJ recombination model characteristic.

To minimise the effort needed to construct a complex set of signal sequences, if needed, we provided simple but efficient python recipes in the documentation of simAIRR. The provided tutorials show how to generate a large set of reference sequences and query them to retrieve a complex set of signal sequences enriched for multiple criteria like the presence of multiple sub-sequence patterns within the sequences (e.g. k-mers) and gene usage. A major advantage of the described approach is that once a large number of reference sequences are generated and stored on disk, they can be queried many times. However, in some cases (e.g. when evaluating a particular behaviour of a developed ML method), one might need to construct signal sequences enriched for very rare sequence patterns. To obtain a sufficiently large number of sequences sharing such rare sequence patterns, one may have to generate and query many millions of sequences. In this manuscript, we did not measure the execution time for mining rare sequence patterns from a set of reference sequences. If the execution time is slow to mine rare sequence patterns, an alternative solution may be to implant sequence patterns (e.g. k-mers) while avoiding the positional biases using existing tools [44] or as we did in our previous study [38]. However, note that the implantation of sequence patterns may lead to another type of shortcut learning opportunities and thus it is important to thoroughly assess the potential pitfalls before using implantation, particularly in benchmarking competitions.

simAIRR currently does not support the simulation of paired chain repertoires and clonal frequencies of receptors in repertoires. Notably, the clonal frequencies of AIRs have been suggested to follow a power law distribution [75]. Future improvements of simAIRR should also simulate clonal frequencies. However, further empirical evidence from independent studies on the usefulness of power law in describing clonal frequency distributions in multiple cell types, subsets and species is needed in this connection. Also note that simAIRR focuses only on simulating datasets for immune state prediction problems at the repertoire-level, but does not focus on receptor-specificity prediction problems.

Conclusion

In summary, the contribution of this study is not only bringing to light the potential shortcut learning opportunity that can arise with the state-of-the-art way of simulating AIRR datasets, but also a novel simulation approach implemented as a Python package that can help avoid potential shortcut learning opportunities for ML methods. Unlike state-of-the-art naive simulation approaches, a key benefit of simAIRR's approach is the possibility of not making any prior assumptions regarding the similarity or commonality of immune state-associated sequences that will be used as true signals. The AIRR datasets simulated using simAIRR were similar to real-world experimental datasets based on the performance of ML methods on both types of datasets. We suggest testing new ML methods on neutral benchmark datasets like simAIRR's to aid unbiased evaluation.

Methods

Outlier measure and likelihood ratio

We computed two different quantitative measures for each public sequence that could potentially aid in the identification of immune state-associated sequences independently. First, an outlier score is computed using a two-step process: (i) given the generation probability (**p_{gen}**) and the average unique number of sequences in a repertoire (number of trials), we computed the probability of observing a public sequence at least once in a repertoire (**p_{obs}**) using the cumulative density function of the binomial distribution. (ii) given the probability of observing a public sequence at least once in a repertoire (**p_{obs}**) and the number of positive labelled repertoires, we further computed the probability of observing a public sequence in the same or higher number of repertoires as it was observed in for a given dataset (**p_{count}**). A negative $\log_{10}$ of p_{count} is referred to as an outlier score throughout the manuscript. Second, a likelihood ratio is computed as the ratio of the empirical probabilities of incidence in positive class examples (repertoires) to negative class examples. Given ***P*** positive class examples and ***N*** negative class examples, **c_P** number of occurrences in positive class examples and **c_N** number of occurrences in negative class examples, the likelihood ratio LR is defined as $\square\square = \frac{\frac{\square\square}{\square}}{\frac{\square\square}{\square}}$.

Empirical relation between generation probability and population incidence

To derive the empirical relation between generation probability and population incidence of sequences from a real-world experimental dataset [18], we first computed the relative frequencies and generation probabilities of each unique sequence including V and J gene masks in a public experimental dataset [18]. For counting the population sequence frequencies, we used CompAIRR [27] and for computing generation probabilities, we used OLGA [47], as described further below. The relative frequencies refer to the proportion of the population carrying a particular sequence. We derived the relation between generation probability and population incidence of sequences separately for the perceived true signal sequences reported by the original study [18] and for all the remaining public sequences. For this, we discretized the entire range of generation probabilities and population incidences into discrete intervals and we placed each unique sequence into its corresponding bin of generation probability and population incidence. With that, we obtained empirical probability distributions describing what fraction of the total unique sequences with a certain generation probability distribution occur at certain population incidence levels in a sample.

Correction of population incidence of public sequences and construction of antigen-experienced-like repertoires

Individuals accumulate immune events over a lifetime. Thus, snapshots of AIRRs, as acquired through targeted immune receptor sequencing from donor blood samples, capture antigen-experienced repertoires that share not only public sequences that are easier to generate, but also other pools of common immune event-associated sequences. However, in silico-generated synthetic repertoires from a method like OLGA behave like naive repertoires that did not experience any immune events. Synthetic naive repertoires share fewer unique sequences and thus tend to carry a lower proportion of public sequences compared to repertoires from experimental datasets. To correct the proportion of public sequences and their population incidence levels, we used the following procedure: we first generate a large number of AIR sequences using the V(D)J recombination model chosen by the user and retain only unique sequences. Notably, the user can choose from any one of the V(D)J recombination models supplied by default with OLGA [47]. We then make a user-desired proportion of sequences public (10% of the sequences is the default option to match experimental datasets). The population incidence

levels (how frequent each unique sequence will be) for the public sequences follows the empirical relation between generation probability and population incidence levels of sequences determined based on a previously published large cohort study of TCR repertoires [18]. As the publicness of sequences can vary between different species, chains, cell type and their subsets, and pairedness of sequences, the users need to calibrate the relation between generation probability and population incidence of sequences when intending to simulate datasets other than human TCRβ chain sequences, which is the default models supplied with simAIRR. The user can supply custom models for the dependence between generation probability and population incidence levels calibrated based on the experimental datasets of their choice.

Assessing the feasibility of a user-desired witness rate

How simAIRR assesses the feasibility of a user-desired witness rate is best explained with an example. For the sake of an illustrative example, we use small numbers for simulation parameters. If the user chooses a pool of 3 sequences as the signal that separates immune-state labels and wants them to be introduced into the positive-labelled repertoires (n=100) of a repertoire dataset (n=200) such that each positively labelled repertoire carries a total of 5 signal sequences on average (desired witness rate), one should be able to introduce a total of 500 instances of the signal (100 x 5) from the pool of 3 sequences. Based on the empirical knowledge of dependence between generation probability and population incidence, if each signal sequence cannot be seen in more than 30% of the total sample size, the pool of 3 sequences together cannot be observed more than 180 times (3 x 200 x 0.3) even if the sequences have a high generation probability. In such a case, it is considered infeasible to meet the user-desired witness rate (of 5 signal sequences per repertoire, amounting to a desired total of 500). simAIRR provides detailed statistics in such a case to help the user in re-configuring the simulation parameters. This could mean that either the user supplies a larger pool of potential signal sequences or modifies the desired witness rate. To avoid expensive computations, users could first use the feasibility assessment mode to make sure that the simulations are feasible given the user-supplied simulation parameters.

Of particular note, the pool of potential signal sequences that one starts with plays a significant role in determining the feasibility of achieving the desired witness rate of signal in the simulated datasets using simAIRR's approach because of the reliance of possible incidence level of sequences on the generation probability of the individual sequences. Thus, unlike in naive simulation approaches, the job of carefully selecting a pool of signal sequences is delegated to the user.

Construction of synthetic baseline repertoires

When constructing synthetic baseline repertoires as a reasonable proxy for real-world experimental repertoires, we ensured the nativeness of the simulated AIR sequences in terms of positional biases, amino acid usage, and sequence length distributions. For this, we generated AIR sequences according to recombination models provided by OLGA [47]. Note that for the analyses of this manuscript, we generated human T cell beta chain receptor datasets, while for simAIRR simulations in general, any of the four default V(D)J models supplied by OLGA [47] (humanTRB, humanTRA, humanIGH, mouseIGH) can be used. Future versions of simAIRR will also allow the usage of user-supplied AIR sequences to construct the synthetic baseline repertoires.

Computation of generation probability

The generation probabilities of AIR sequences are computed using OLGA [47] with the default generative models of the V(D)J recombination model while including both the masks for V and J genes

for each sequence. For the analyses of this manuscript, we computed the generation probabilities using the default V(D)J recombination model of the human T cell beta chain receptor. Notably, any of the four default V(D)J recombination models supplied with OLGA [47] can be used as mentioned above.

ML models, training, selection and evaluation

We used two different ML methods in the case studies. In the first case study, the signal sequences share any of the three chosen 4-mers. For those datasets, where the signal can be captured by 4-mers, we used an ML method that matches with the described at length in our previous study [38]. Briefly, we used a highly-regularized logistic regression model on a 4-mer encoded representation of the amino acid sequences. The hyperparameters for the model were chosen through nested cross-validation. For details on the implementations and hyperparameter optimizations, see relevant descriptions in [38]. In the second case study, we did not have prior knowledge of the sequence similarity patterns of the signal sequences. Therefore, we used an ML method that assumes the full sequence identity as a potential signal representation [18]. Specifically, we used a probabilistic binary classifier based on phenotype burden [18] implemented in immuneML [37]. We used five-fold nested cross-validation and an exhaustive grid search for hyperparameter optimization as in our previous study [38] for both ML methods. Balanced accuracy was used as the performance metric for optimization during training and the ROC AUC was reported for the sake of comparison with previous studies that used the same metric.

Querying sequences enriched for k-mer-like patterns

In the first case study, we assumed that the true signal sequences share a similarity in terms of shared k-mers. Specifically, the signal sequences were required to carry any one of the three chosen k-mers: *WKDY, YREV, and ERFY.* In our previous study [38], we implanted k-mers in the central portion of the CDR3 amino acid sequences to obtain such signal sequences. However, the implantation of k-mers can introduce additional artefacts by destroying the biological properties of the sequence, which the ML methods can exploit as another way of shortcut learning. To avoid that, in this study, we queried a large set of reference sequences to retrieve all those sequences that carry a k-mer of interest. To make such pattern matching process easy for the users if need be, we provide a simple python script based on the bionumpy library [76] and a corresponding tutorial that shows how to generate a large set of reference sequences and retrieve the pattern-matched sequences of interest with minimal effort and with very few lines of Python code [60]. We also provided corresponding examples using Unix grep. In the recipe, we have shown how to construct a complex set of signal sequences enriched for multiple criteria like the presence of multiple sub-sequence patterns within the sequences and gene usage. The code recipes not only require a minimal coding effort but are also efficient and have a minimal run-time. For instance, the wall time for generating 10 million sequences when using 40 processes was less than one minute. Similarly, the execution wall time for querying the 10 million sequences for selected k-mer patterns was less than one minute. Note that the query time can increase with the number of queried patterns for any pattern-matching tool including Unix grep. A key benefit of the approach described here is that once a large number of reference sequences are generated and stored on disk, they can be queried multiple times.

Customisable simulation parameters

All the simulation parameters of simAIRR are customisable. Some of the key customisable parameters include one of the four possible V(D)J recombination models, the number of repertoires and the proportion of positive class-labelled repertoires, average sequencing depth, the proportion of public

sequences and witness rate. In addition, the users are required to supply a pool of sequences that will be considered true signal sequences. It is also possible to control the number of sequences that will be used as a true signal.

Docker container to improve reproducibility

To ease the installation issues, allow quick testing and improve portability, we supply a Docker image [77] with a predefined computing environment maintaining all the dependencies required for the execution of simAIRR workflows with minimal overhead. The docker image is hosted on DockerHub and can be accessed at *kanduric/simairr:latest.*

Graphics

ggplot2 version 3.3.6 [78] was used for graphs and Inkscape version 1.0.1 [79] was used for illustrations.

Data and Source Code availability

- Source code of simAIRR: https://github.com/KanduriC/simAIRR
- Snapshot of the frozen codebase with permanent DOI: https://doi.org/10.5281/zenodo.7534810.
- Docker image at docker hub: *kanduric/simairr:latest*
- Configuration files to reproduce the simulations and ML models of the use cases: https://github.com/KanduriC/usecases_simairr.git
- Simulated datasets used in the case studies with permanent DOI: https://doi.org/10.11582/2023.00011.
- A simple python recipe and tutorial for generating sequences enriched for k-mer-like sequence patterns to be used as true signal: https://kanduric.github.io/simAIRR/tutorials.html#querying-sequences-enriched-for-k-mer-like-patterns

Availability of source code and requirements

Project name: simAIRR
Project home page: https://github.com/KanduriC/simAIRR
Operating system(s): Platform independent
Programming language: Python
Other requirements: Python 3.8 or higher
License: GNU AGPL version 3.

List of abbreviations

AIR	Adaptive Immune Receptor
AIRR	Adaptive Immune Receptor Repertoire
BCR	B-Cell Receptors
CDR3	Complementarity Determining Region 3
HCV	Hepatitis C virus

IMGT	ImMunoGeneTics
ML	Machine Learning
TCR	T-Cell Receptors
TCRβ	T-Cell Receptor beta chain

Ethics approval and Consent

Not applicable

Consent for publication

Not applicable

Competing interests

V.G. declares advisory board positions in aiNET GmbH, Enpicom B.V, Absci, Omniscope, and Diagonal Therapeutics. VG is a consultant for Adaptyv Biosystems, Specifica Inc, Roche/Genentech, immunai, and LabGenius.

Funding

The Leona M. and Harry B. Helmsley Charitable Trust (#2019PG-T1D011, to VG), UiO World-Leading Research Community (to VG), UiO: LifeScience Convergence Environment Immunolingo (to VG and GKS), EU Horizon 2020 iReceptorplus (#825821) (to VG), a Norwegian Cancer Society Grant (#215817, to VG), a Research Council of Norway projects (#300740, #331890 to VG), a Research Council of Norway IKTPLUSS project (#311341, to VG and GKS), and Stiftelsen Kristian Gerhard Jebsen (K.G. Jebsen Coeliac Disease Research Centre) (to GKS).

Authors' contributions

CK and GKS conceived the overall study. CK designed and developed the code, performed analyses and drafted the manuscript. LS and MP contributed with ML method implementations in immuneML [37] that were used in the case studies. KR contributed with the bionumpy [76] recipe [60] for retrieving sequences enriched for sequence patterns. MC, OP, GY, and VG participated in brainstorming and provided critical conceptual feedback. All authors read, provided critical edits and approved the final manuscript.

Acknowledgements

Some of the analyses in this work were performed using the Immunohub eInfrastructure funded by the University of Oslo and operated by the authors in close collaboration with the University Senter for Information Technology (USIT), University of Oslo.

References

1. Calis JJA, Rosenberg BR. Characterizing immune repertoires by high throughput sequencing: strategies and applications. *Trends Immunol*. 2014; doi: 10.1016/j.it.2014.09.004.

2. Georgiou G, Ippolito GC, Beausang J, Busse CE, Wardemann H, Quake SR. The promise and challenge of high-throughput sequencing of the antibody repertoire. *Nat Biotechnol*.

2014; doi: 10.1038/nbt.2782.
3. Arnaout RA, Prak ETL, Schwab N, Rubelt F, Adaptive Immune Receptor Repertoire Community. The Future of Blood Testing Is the Immunome. *Front Immunol*. 2021; doi: 10.3389/fimmu.2021.626793.
4. Miho E, Yermanos A, Weber CR, Berger CT, Reddy ST, Greiff V. Computational Strategies for Dissecting the High-Dimensional Complexity of Adaptive Immune Repertoires. *Front Immunol*. 2018; doi: 10.3389/fimmu.2018.00224.
5. Greiff V, Yaari G, Cowell LG. Mining adaptive immune receptor repertoires for biological and clinical information using machine learning. *Curr Opin Syst Biol*. 2020; doi: https://doi.org/10.1016/j.coisb.2020.10.010.
6. Venturi V, Price DA, Douek DC, Davenport MP. The molecular basis for public T-cell responses? *Nat Rev Immunol*. 2008; doi: 10.1038/nri2260.
7. Li H, Ye C, Ji G, Han J. Determinants of public T cell responses. *Cell Res*. 2012; doi: 10.1038/cr.2012.1.
8. Madi A, Shifrut E, Reich-Zeliger S, Gal H, Best K, Ndifon W, et al.. T-cell receptor repertoires share a restricted set of public and abundant CDR3 sequences that are associated with self-related immunity. *Genome Res*. 2014; doi: 10.1101/gr.170753.113.
9. Madi A, Poran A, Shifrut E, Reich-Zeliger S, Greenstein E, Zaretsky I, et al.. T cell receptor repertoires of mice and humans are clustered in similarity networks around conserved public CDR3 sequences. *eLife*. 2017; doi: 10.7554/eLife.22057.
10. Pogorelyy MV, Elhanati Y, Marcou Q, Sycheva AL, Komech EA, Nazarov VI, et al.. Persisting fetal clonotypes influence the structure and overlap of adult human T cell receptor repertoires. *PLoS Comput Biol*. 2017; doi: 10.1371/journal.pcbi.1005572.
11. Greiff V, Menzel U, Miho E, Weber C, Riedel R, Cook S, et al.. Systems Analysis Reveals High Genetic and Antigen-Driven Predetermination of Antibody Repertoires throughout B Cell Development. *Cell Rep*. 2017; doi: 10.1016/j.celrep.2017.04.054.
12. Trück J, Ramasamy MN, Galson JD, Rance R, Parkhill J, Lunter G, et al.. Identification of antigen-specific B cell receptor sequences using public repertoire analysis. *J Immunol Baltim Md 1950*. 2015; doi: 10.4049/jimmunol.1401405.
13. Jaffe DB, Shahi P, Adams BA, Chrisman AM, Finnegan PM, Raman N, et al.. Functional antibodies exhibit light chain coherence. *Nature*. 2022; doi: 10.1038/s41586-022-05371-z.
14. Pertseva M, Gao B, Neumeier D, Yermanos A, Reddy ST. Applications of Machine and Deep Learning in Adaptive Immunity. *Annu Rev Chem Biomol Eng*. 2021; doi: 10.1146/annurev-chembioeng-101420-125021.
15. Katayama Y, Yokota R, Akiyama T, Kobayashi TJ. Machine Learning Approaches to TCR Repertoire Analysis. *Front Immunol*. 132022;
16. Glanville J, Huang H, Nau A, Hatton O, Wagar LE, Rubelt F, et al.. Identifying specificity groups in the T cell receptor repertoire. *Nature*. 2017; doi: 10.1038/nature22976.
17. Dash P, Fiore-Gartland AJ, Hertz T, Wang GC, Sharma S, Souquette A, et al.. Quantifiable predictive features define epitope specific T cell receptor repertoires. *Nature*. 2017; doi: 10.1038/nature22383.
18. Emerson RO, DeWitt WS, Vignali M, Gravley J, Hu JK, Osborne EJ, et al.. Immunosequencing identifies signatures of cytomegalovirus exposure history and HLA-mediated effects on the T cell repertoire. *Nat Genet*. 2017; doi: 10.1038/ng.3822.
19. Pogorelyy MV, Minervina AA, Chudakov DM, Mamedov IZ, Lebedev YB, Mora T, et al.. Method for identification of condition-associated public antigen receptor sequences. *eLife*. 2018; doi: 10.7554/eLife.33050.
20. Ostmeyer J, Christley S, Toby IT, Cowell LG. Biophysicochemical Motifs in T-cell

Receptor Sequences Distinguish Repertoires from Tumor-Infiltrating Lymphocyte and Adjacent Healthy Tissue. *Cancer Res*. 2019; doi: 10.1158/0008-5472.CAN-18-2292.
21. Zhang H, Liu L, Zhang J, Chen J, Ye J, Shukla S, et al.. Investigation of Antigen-Specific T-Cell Receptor Clusters in Human Cancers. *Clin Cancer Res Off J Am Assoc Cancer Res*. 2020; doi: 10.1158/1078-0432.CCR-19-3249.
22. Huang H, Wang C, Rubelt F, Scriba TJ, Davis MM. Analyzing the Mycobacterium tuberculosis immune response by T-cell receptor clustering with GLIPH2 and genome-wide antigen screening. *Nat Biotechnol*. 2020; doi: 10.1038/s41587-020-0505-4.
23. Widrich M, Schäfl B, Pavlović M, Ramsauer H, Gruber L, Holzleitner M, et al.. Modern Hopfield Networks and Attention for Immune Repertoire Classification. *bioRxiv*. 2020; doi: 10.1101/2020.04.12.038158.
24. Beshnova D, Ye J, Onabolu O, Moon B, Zheng W, Fu Y-X, et al.. De novo prediction of cancer-associated T cell receptors for noninvasive cancer detection. *Sci Transl Med*. 2020; doi: 10.1126/scitranslmed.aaz3738.
25. Shemesh O, Polak P, Lundin KEA, Sollid LM, Yaari G. Machine Learning Analysis of Naïve B-Cell Receptor Repertoires Stratifies Celiac Disease Patients and Controls. *Front Immunol*. 2021; doi: 10.3389/fimmu.2021.627813.
26. Sidhom J-W, Larman HB, Pardoll DM, Baras AS. DeepTCR is a deep learning framework for revealing sequence concepts within T-cell repertoires. *Nat Commun*. 2021; doi: 10.1038/s41467-021-21879-w.
27. Rognes T, Scheffer L, Greiff V, Sandve GK. CompAIRR: ultra-fast comparison of adaptive immune receptor repertoires by exact and approximate sequence matching. bioRxiv;
28. Greiff V, Weber CR, Palme J, Bodenhofer U, Miho E, Menzel U, et al.. Learning the High-Dimensional Immunogenomic Features That Predict Public and Private Antibody Repertoires. *J Immunol*. 2017; doi: 10.4049/jimmunol.1700594.
29. Akbar R, Robert PA, Pavlović M, Jeliazkov JR, Snapkov I, Slabodkin A, et al.. A compact vocabulary of paratope-epitope interactions enables predictability of antibody-antigen binding. *Cell Rep*. 2021; doi: 10.1016/j.celrep.2021.108856.
30. Pogorelyy MV, Minervina AA, Shugay M, Chudakov DM, Lebedev YB, Mora T, et al.. Detecting T cell receptors involved in immune responses from single repertoire snapshots. *PLoS Biol*. 2019; doi: 10.1371/journal.pbio.3000314.
31. Jokinen E, Huuhtanen J, Mustjoki S, Heinonen M, Lähdesmäki H. Predicting recognition between T cell receptors and epitopes with TCRGP. *PLoS Comput Biol*. 2021; doi: 10.1371/journal.pcbi.1008814.
32. Robert PA, Akbar R, Frank R, Pavlović M, Widrich M, Snapkov I, et al.. Unconstrained generation of synthetic antibody–antigen structures to guide machine learning methodology for antibody specificity prediction. *Nat Comput Sci*. Nature Publishing Group; 2022; doi: 10.1038/s43588-022-00372-4.
33. Greiff V, Bhat P, Cook SC, Menzel U, Kang W, Reddy ST. A bioinformatic framework for immune repertoire diversity profiling enables detection of immunological status. *Genome Med*. 2015; doi: 10.1186/s13073-015-0169-8.
34. Sidhom J-W, Oliveira G, Ross-MacDonald P, Wind-Rotolo M, Wu CJ, Pardoll DM, et al.. Deep learning reveals predictive sequence concepts within immune repertoires to immunotherapy. *Sci Adv*. 2022; doi: 10.1126/sciadv.abq5089.
35. Xu JL, Davis MM. Diversity in the CDR3 region of V(H) is sufficient for most antibody specificities. *Immunity*. 2000; doi: 10.1016/s1074-7613(00)00006-6.
36. Rudolph MG, Stanfield RL, Wilson IA. How TCRs bind MHCs, peptides, and coreceptors.

Annu Rev Immunol. 2006; doi: 10.1146/annurev.immunol.23.021704.115658.
37. Pavlović M, Scheffer L, Motwani K, Kanduri C, Kompova R, Vazov N, et al.. The immuneML ecosystem for machine learning analysis of adaptive immune receptor repertoires. *Nat Mach Intell*. 2021; doi: 10.1038/s42256-021-00413-z.
38. Kanduri C, Pavlović M, Scheffer L, Motwani K, Chernigovskaya M, Greiff V, et al.. Profiling the baseline performance and limits of machine learning models for adaptive immune receptor repertoire classification. *GigaScience*. 2022; doi: 10.1093/gigascience/giac046.
39. Sandve GK, Greiff V. Access to ground truth at unconstrained size makes simulated data as indispensable as experimental data for bioinformatics methods development and benchmarking. *Bioinforma Oxf Engl*. 2022; doi: 10.1093/bioinformatics/btac612.
40. Chen V, Yang M, Cui W, Kim JS, Talwalkar A, Ma J. Best Practices for Interpretable Machine Learning in Computational Biology. bioRxiv;
41. Liu X, Zhang W, Zhao M, Fu L, Liu L, Wu J, et al.. T cell receptor β repertoires as novel diagnostic markers for systemic lupus erythematosus and rheumatoid arthritis. *Ann Rheum Dis*. 2019; doi: 10.1136/annrheumdis-2019-215442.
42. Nolan S, Vignali M, Klinger M, Dines JN, Kaplan IM, Svejnoha E, et al.. A large-scale database of T-cell receptor beta (TCRβ) sequences and binding associations from natural and synthetic exposure to SARS-CoV-2. *Res Sq*. 2020; doi: 10.21203/rs.3.rs-51964/v1.
43. Zaslavsky ME, Ram-Mohan N, Guthridge JM, Merrill JT, Goldman JD, Lee J-Y, et al.. Disease diagnostics using machine learning of immune receptors. bioRxiv;
44. Weber CR, Akbar R, Yermanos A, Pavlović M, Snapkov I, Sandve GK, et al.. immuneSIM: tunable multi-feature simulation of B- and T-cell receptor repertoires for immunoinformatics benchmarking. *Bioinformatics*. 2020; doi: 10.1093/bioinformatics/btaa158.
45. Sutherland C, Cowan GJM. AIRRSHIP: simulating human B cell receptor repertoire sequences. bioRxiv;
46. Safonova Y, Lapidus A, Lill J. IgSimulator: a versatile immunosequencing simulator. *Bioinforma Oxf Engl*. 2015; doi: 10.1093/bioinformatics/btv326.
47. Sethna Z, Elhanati Y, Callan CG, Walczak AM, Mora T. OLGA: fast computation of generation probabilities of B- and T-cell receptor amino acid sequences and motifs. *Bioinformatics*. 2019; doi: 10.1093/bioinformatics/btz035.
48. Marcou Q, Mora T, Walczak AM. High-throughput immune repertoire analysis with IGoR. *Nat Commun*. 2018; doi: 10.1038/s41467-018-02832-w.
49. Geirhos R, Jacobsen J-H, Michaelis C, Zemel R, Brendel W, Bethge M, et al.. Shortcut Learning in Deep Neural Networks. *Nat Mach Intell*. 2020; doi: 10.1038/s42256-020-00257-z.
50. Davis MM, Bjorkman PJ. T-cell antigen receptor genes and T-cell recognition. *Nature*. 1988; doi: 10.1038/334395a0.
51. Tonegawa S. Somatic generation of antibody diversity. *Nature*. 1983; doi: 10.1038/302575a0.
52. Fazilleau N, Cabaniols J-P, Lemaître F, Motta I, Kourilsky P, Kanellopoulos JM. Valpha and Vbeta public repertoires are highly conserved in terminal deoxynucleotidyl transferase-deficient mice. *J Immunol Baltim Md 1950*. 2005; doi: 10.4049/jimmunol.174.1.345.
53. Venturi V, Kedzierska K, Price DA, Doherty PC, Douek DC, Turner SJ, et al.. Sharing of T cell receptors in antigen-specific responses is driven by convergent recombination. *Proc Natl Acad Sci U S A*. 2006; doi: 10.1073/pnas.0608907103.
54. Quigley MF, Greenaway HY, Venturi V, Lindsay R, Quinn KM, Seder RA, et al..

Convergent recombination shapes the clonotypic landscape of the naïve T-cell repertoire. *Proc Natl Acad Sci U S A*. 2010; doi: 10.1073/pnas.1010586107.
55. Venturi V, Quigley MF, Greenaway HY, Ng PC, Ende ZS, McIntosh T, et al.. A mechanism for TCR sharing between T cell subsets and individuals revealed by pyrosequencing. *J Immunol Baltim Md 1950*. 2011; doi: 10.4049/jimmunol.1003898.
56. Shugay M, Bagaev DV, Zvyagin IV, Vroomans RM, Crawford JC, Dolton G, et al.. VDJdb: a curated database of T-cell receptor sequences with known antigen specificity. *Nucleic Acids Res*. 2018; doi: 10.1093/nar/gkx760.
57. Elhanati Y, Sethna Z, Callan CG, Mora T, Walczak AM. Predicting the spectrum of TCR repertoire sharing with a data- driven model of recombination. *Immunol Rev*. 2018; doi: 10.1111/imr.12665.
58. Shugay M, Bolotin DA, Putintseva EV, Pogorelyy MV, Mamedov IZ, Chudakov DM. Huge Overlap of Individual TCR Beta Repertoires. *Front Immunol*. 2013; doi: 10.3389/fimmu.2013.00466.
59. Murugan A, Mora T, Walczak AM, Callan CG. Statistical inference of the generation probability of T-cell receptors from sequence repertoires. *Proc Natl Acad Sci U S A*. 2012; doi: 10.1073/pnas.1212755109.
60. : Tutorials — simAIRR documentation. https://kanduric.github.io/simAIRR/tutorials.html#querying-sequences-enriched-for-k-mer-like-patterns Accessed 2023 Jan 11.
61. Katayama Y, Kobayashi TJ. Comparative Study of Repertoire Classification Methods Reveals Data Efficiency of k -mer Feature Extraction. *Front Immunol*. 2022; doi: 10.3389/fimmu.2022.797640.
62. Kaufman S, Rosset S, Perlich C, Stitelman O. Leakage in data mining: Formulation, detection, and avoidance. *ACM Trans Knowl Discov Data*. 2012; doi: 10.1145/2382577.2382579.
63. Kapoor S, Narayanan A. Leakage and the Reproducibility Crisis in ML-based Science. arXiv;
64. Tickotsky N, Sagiv T, Prilusky J, Shifrut E, Friedman N. McPAS-TCR: a manually curated catalogue of pathology-associated T cell receptor sequences. *Bioinforma Oxf Engl*. 2017; doi: 10.1093/bioinformatics/btx286.
65. Fleri W, Paul S, Dhanda SK, Mahajan S, Xu X, Peters B, et al.. The Immune Epitope Database and Analysis Resource in Epitope Discovery and Synthetic Vaccine Design. *Front Immunol*. 2017; doi: 10.3389/fimmu.2017.00278.
66. Cinelli M, Sun Y, Best K, Heather JM, Reich-Zeliger S, Shifrut E, et al.. Feature selection using a one dimensional naïve Bayes' classifier increases the accuracy of support vector machine classification of CDR3 repertoires. *Bioinformatics*. 2017; doi: 10.1093/bioinformatics/btw771.
67. Sun Y, Best K, Cinelli M, Heather JM, Reich-Zeliger S, Shifrut E, et al.. Specificity, Privacy, and Degeneracy in the CD4 T Cell Receptor Repertoire Following Immunization. *Front Immunol*. 2017; doi: 10.3389/fimmu.2017.00430.
68. Thomas N, Best K, Cinelli M, Reich-Zeliger S, Gal H, Shifrut E, et al.. Tracking global changes induced in the CD4 T-cell receptor repertoire by immunization with a complex antigen using short stretches of CDR3 protein sequence. *Bioinformatics*. 2014; doi: 10.1093/bioinformatics/btu523.
69. De Neuter N, Bartholomeus E, Elias G, Keersmaekers N, Suls A, Jansens H, et al.. Memory CD4+ T cell receptor repertoire data mining as a tool for identifying cytomegalovirus serostatus. *Genes Immun*. 2019; doi: 10.1038/s41435-018-0035-y.

70. Boulesteix A-L, Lauer S, Eugster MJA. A plea for neutral comparison studies in computational sciences. *PloS One*. 2013; doi: 10.1371/journal.pone.0061562.
71. Weber LM, Saelens W, Cannoodt R, Soneson C, Hapfelmeier A, Gardner PP, et al.. Essential guidelines for computational method benchmarking. *Genome Biol*. 2019; doi: 10.1186/s13059-019-1738-8.
72. Slabodkin A, Chernigovskaya M, Mikocziova I, Akbar R, Scheffer L, Pavlović M, et al.. Individualized VDJ recombination predisposes the available Ig sequence space. *Genome Res*. 2021; doi: 10.1101/gr.275373.121.
73. Elhanati Y, Sethna Z, Marcou Q, Callan CG, Mora T, Walczak AM. Inferring processes underlying B-cell repertoire diversity. *Philos Trans R Soc Lond B Biol Sci*. 2015; doi: 10.1098/rstb.2014.0243.
74. Sethna Z, Isacchini G, Dupic T, Mora T, Walczak AM, Elhanati Y. Population variability in the generation and selection of T-cell repertoires. *PLoS Comput Biol*. 2020; doi: 10.1371/journal.pcbi.1008394.
75. Desponds J, Mora T, Walczak AM. Fluctuating fitness shapes the clone-size distribution of immune repertoires. *Proc Natl Acad Sci*. Proceedings of the National Academy of Sciences; 2016; doi: 10.1073/pnas.1512977112.
76. Rand K, Grytten I, Pavlovic M, Kanduri C, Sandve GK. BioNumPy: Fast and easy analysis of biological data with Python. bioRxiv;
77. : kanduric/simairr - Docker Image | Docker Hub. https://hub.docker.com/r/kanduric/simairr Accessed 2023 Jan 13.
78. Wickham H. ggplot2: Elegant Graphics for Data Analysis. Springer-Verlag New York;
79. Inkscape Project. Inkscape.
