# Supplementary material for: simAIRR: simulation of adaptive immune repertoires with realistic receptor sequence sharing for benchmarking of immune state prediction methods
Source: Gigascience. 2023 Oct 17;12:giad074. doi: 10.1093/gigascience/giad074 (PMC10580376; doi:10.1093/gigascience/giad074)
Supplement: giad074_Supplemental_File [file giad074_supplemental_file.docx]

**Supplementary Information**

**simAIRR: simulation of adaptive immune repertoires with realistic receptor sequence sharing for benchmarking of immune state prediction methods**

Chakravarthi Kanduri^1,2,#^ ([skanduri@uio.no](mailto:skanduri@uio.no)), Lonneke Scheffer^1^ ([lonnekes@ifi.uio.no](mailto:lonnekes@ifi.uio.no)), Milena Pavlović^1,2^  ([milenpa@student.matnat.uio.no](mailto:milenpa@student.matnat.uio.no)), Knut Dagestad Rand^1^ ([knutdr@math.uio.no](mailto:knutdr@math.uio.no)), Maria Chernigovskaya^3^ ([mariia.chernigovskaya@medisin.uio.no](mailto:mariia.chernigovskaya@medisin.uio.no)), Oz Pirvandy^4^ ([pirvandy@gmail.com](mailto:pirvandy@gmail.com)), Gur Yaari^4^ ([gur.yaari@biu.ac.il](mailto:gur.yaari@biu.ac.il)), Victor Greiff^3^ ([victor.greiff@medisin.uio.no](mailto:victor.greiff@medisin.uio.no)), Geir K. Sandve^1,2,#^ ([geirksa@ifi.uio.no](mailto:geirksa@ifi.uio.no))

^1^ Centre for Bioinformatics, Department of Informatics, University of Oslo, Norway

^2^ UiORealArt Convergence Environment, University of Oslo, Norway

^3^ Department of Immunology and Oslo University Hospital, University of Oslo, Norway

^4^ Faculty of Engineering, Bar-Ilan University, Israel

^#^Correspondence


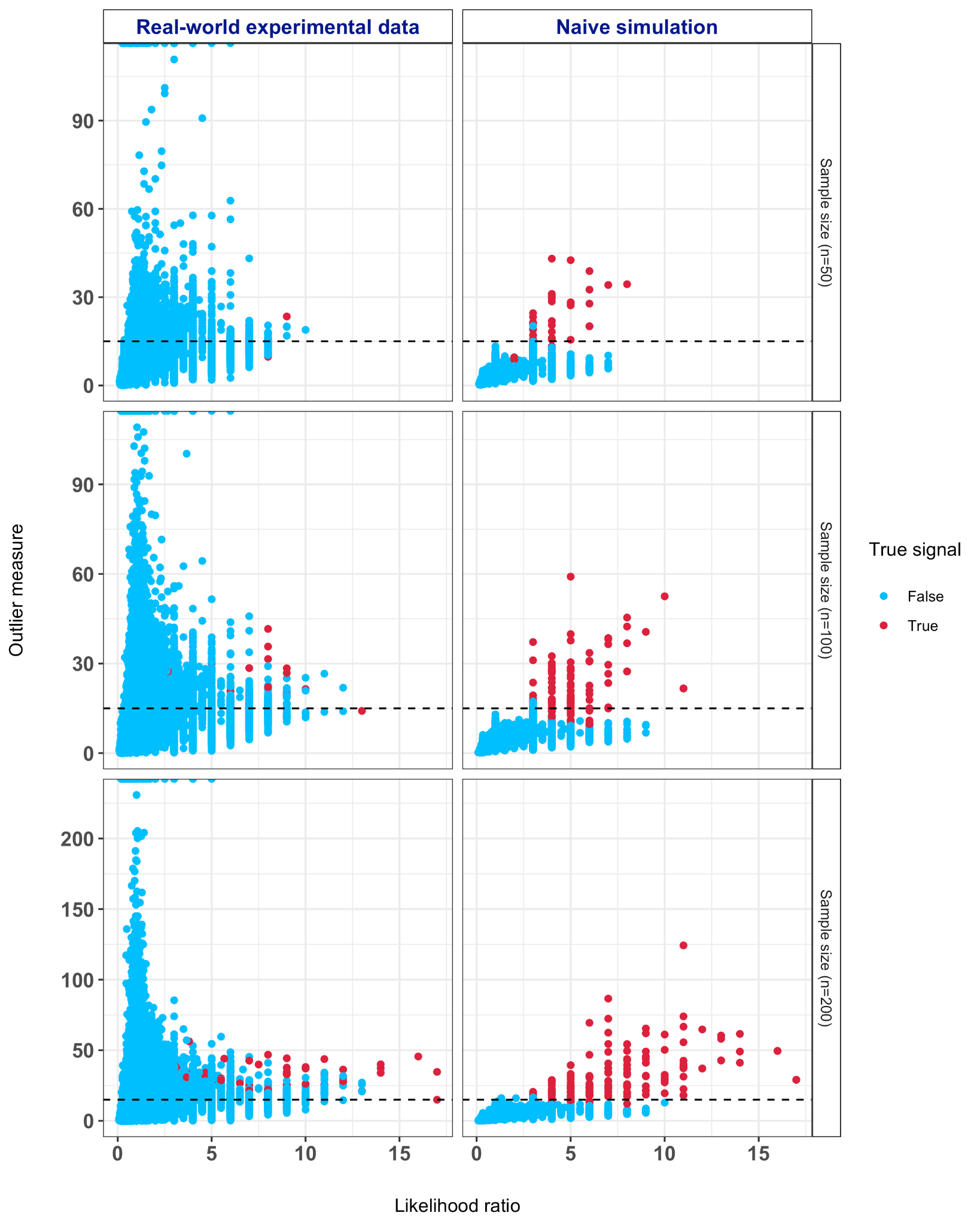


**Figure S1: Generation probability discordance bias in the naive simulation of AIRR datasets at different sample sizes.** We randomly sub-sampled repertoires of different sample sizes (n=50, 100, 200) with balanced labels from a real-world experimental AIRR dataset (n=683 repertoires; 307 positive class examples and 376 negative class examples, average unique number of TCRβs per repertoire ~ 200k) [[1]](https://www.zotero.org/google-docs/?ofou91) and repeated the experiments shown in Figure 2 of the main text. For comparison, we also included similar-sized data from naive simulations. The findings at different sample sizes are shown in different rows of the chart. Similar to the observations of Figure 2, the outlier measure (y-axis) alone is a poor classifier of presumed true signals even in the sub-sampled real-world experimental data at different sample sizes (charts on the left column). On the contrary, the outlier measure alone has very high precision in synthetic datasets generated through the naive simulation approach described in Figure 1.b.ii of the main text.


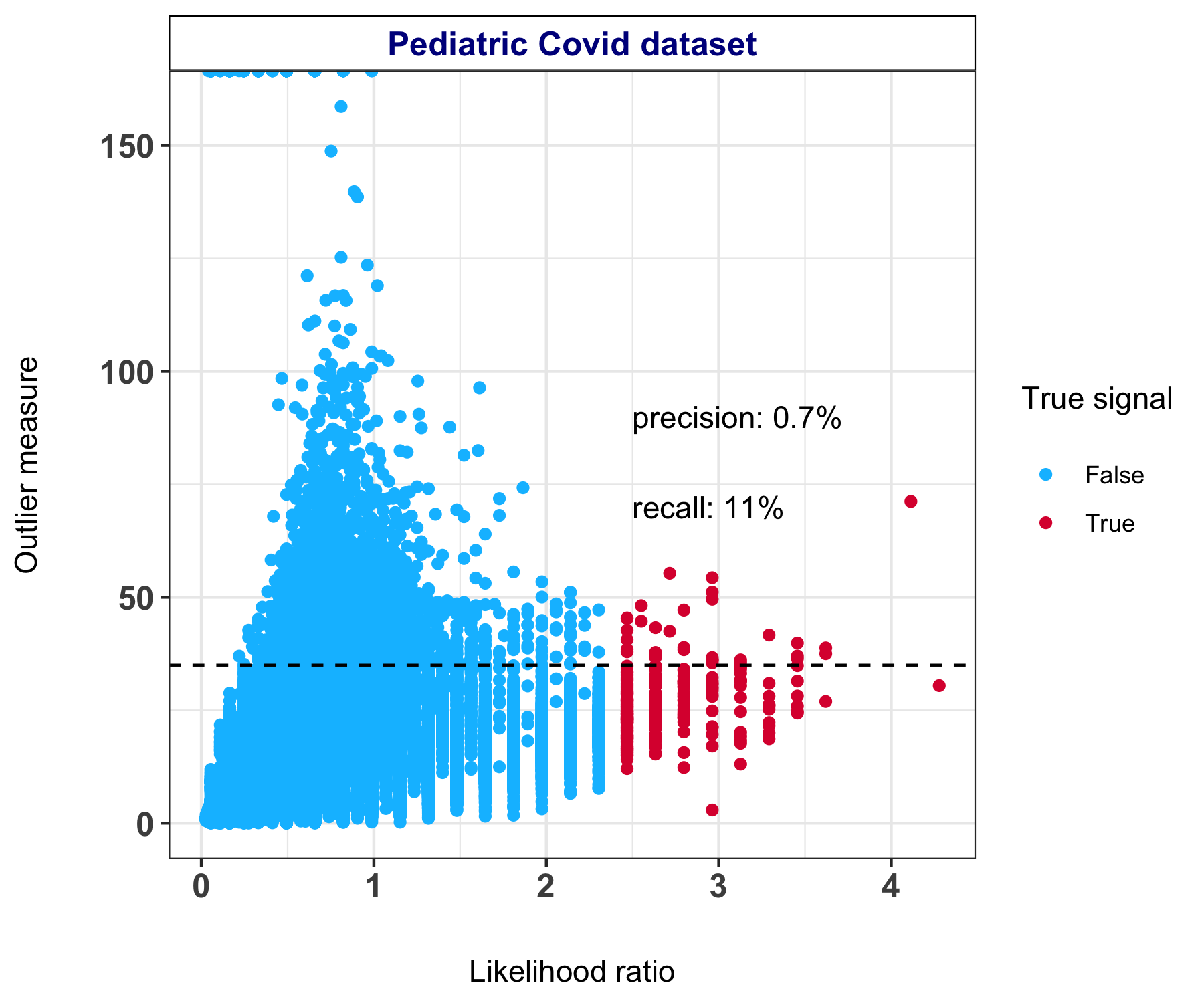


**Figure S2: Absence of generation probability discordance bias in a real-world experimental dataset of a small sample size.** The phenomenon of the absence of generation probability discordance bias in real-world experimental data was also observed on another TCRB dataset with a much smaller sample size (compared to [[1]](https://www.zotero.org/google-docs/?1Zhkar)) of 79 cases and 13 controls with/without pediatric Covid-19 from [[2]](https://www.zotero.org/google-docs/?VOqV0S). Sacco et al. assayed TCRB data from pediatric Covid-19 patients that either progressed to develop multisystem inflammatory syndrome or not (together 79 cases) and matched healthy controls (13 controls). In the original article of Sacco et al., TCRB data of Covid-19 positive cases were not compared against healthy controls to identify potential public clones. However, here, for the sake of investigating the presence/absence of generation probability discordance bias in a dataset of as large a sample size as possible, we included all 79 cases and 13 controls in the analysis. Since the condition-associated public clones are not reported in the original study, we looked at the distribution of likelihood ratio (on the x-axis) and placed an arbitrary threshold to call sequences as presumed signals. Irrespective of the presumed signal sequences, the main observation from this analysis is that similar to the observations of Figure 2 and Supplementary Figure S1, in the real-world experimental datasets, many public sequences are observed with increased population incidence than what is expected based on their generation probability. In other words, thresholding based on the computed outlier score alone will be a poor classifier of signal sequences.


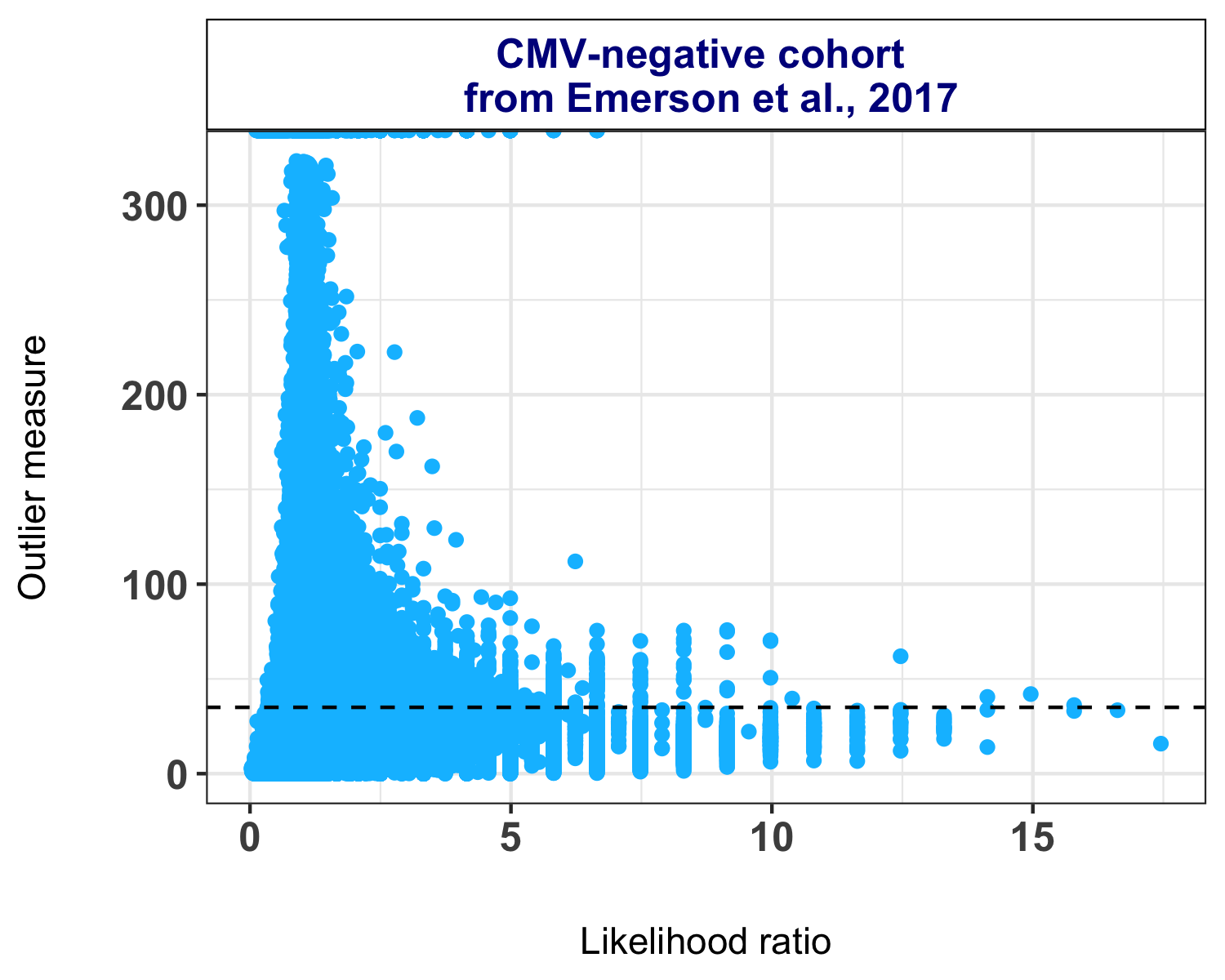


**Figure S3: Presence of many receptor sequences at an unlikely high population incidence than what is expected based on their generation probability in the CMV-negative cohort of Emerson et al., 2017.** We used the real-world experimental AIRR dataset with CMV-positive and CMV-negative repertoires that were used for the analyses in Figure 2.a (n=683 repertoires; 307 CMV-positive examples and 376 CMV-negative examples, average unique number of TCRβs per repertoire ~ 200k). Instead of computing the outlier measure (y-axis) based on CMV-positive examples, we repeated the same experiment on CMV-negative cohort as a control experiment to demonstrate that antigen-experienced repertoires carry many outlier sequences that occur at an unlikely high population incidence than what is expected based on their generation probability. Similar to the observations of Figure 2.a, even in the CMV-negative cohort, many sequences were observed with high outlier scores (on the y-axis). Here, each point represents a unique TCRβ CDR3 sequence. When computing the likelihood ratio (on the x-axis) as described in the methods section of the main manuscript, we treated the CMV-negative examples as a positive class and used the CMV-positive cohort as a negative class. The data points in red as shown in Figure 2 are absent in this figure because we did not consider any sequences as condition-associated or true signals. Nevertheless, the main message from this analysis is the observation of many AIR sequences in antigen-experienced repertoires that occur at an unlikely population incidence level than what is expected given their generation probability as measured through the outlier score on the y-axis.


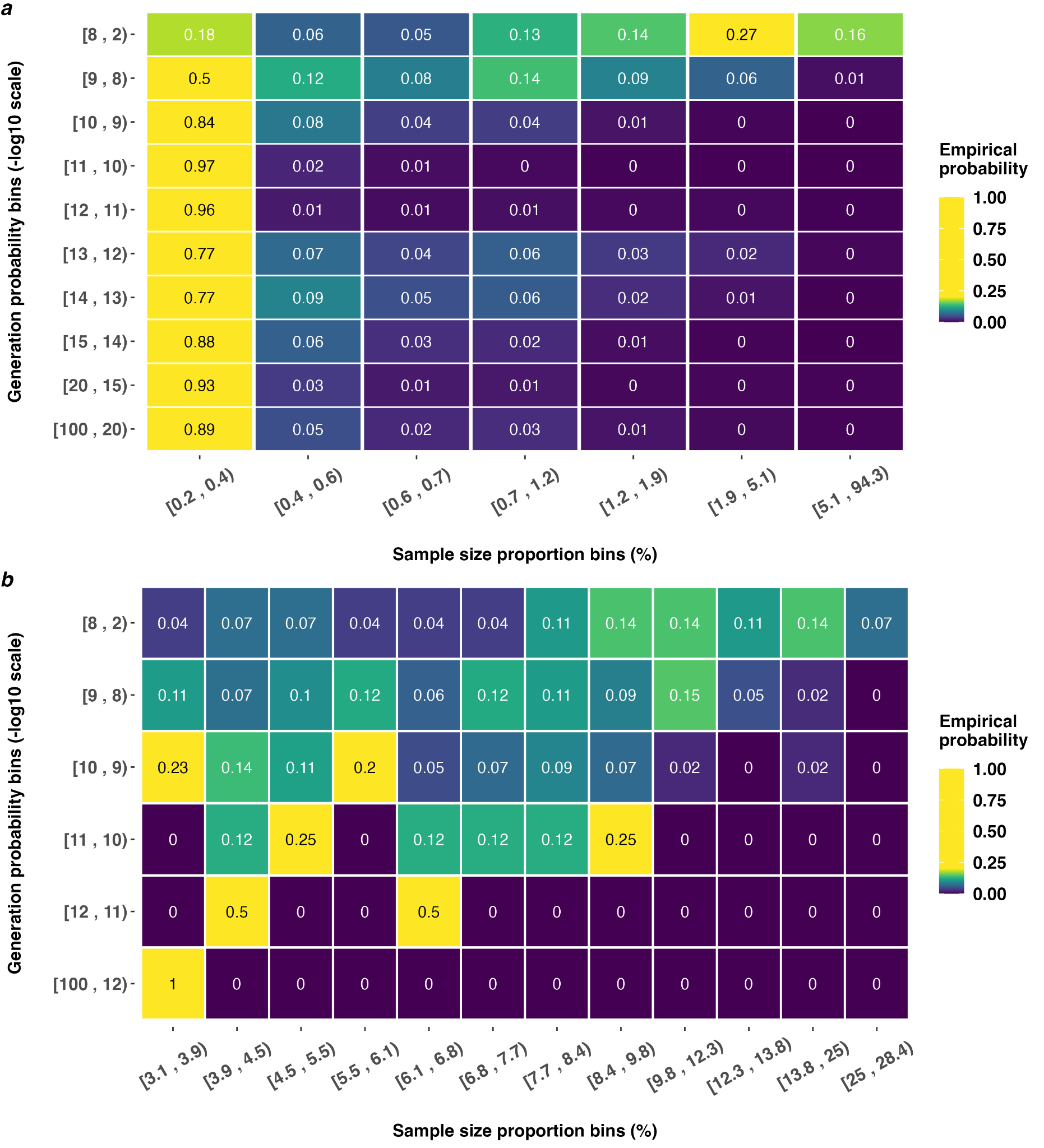


**Figure S4: Empirical relation between generation probability and population incidence of public AIR sequences.** The relation between generation probability and population incidence of public AIR sequences was determined based on a previously published large cohort study of TCR repertoires [[1]](https://www.zotero.org/google-docs/?RoNV7V) separately for signal (**3.b**) and all the remaining public sequences (**3.a**)**.** In both **a** and **b,** the x-axis represents the sample size proportion bins and the y-axis represents generation probability distribution bins in the -log10 scale. Note that the bins on both axes are half-open intervals that include the left endpoint but exclude the right endpoint. To explain one cell of the heatmaps, consider the top-left cell in panel **a**. The cell corresponding to the generation probability bin [8, 2) and sample size proportion bin [0.2, 0.4) tell that 18% of the total unique public sequences with a generation probability between [8, 2) (in -log10 scale) occur between [0.2, 0.4) % of total repertoires in the sample (dataset). The row-sums of the heatmaps should sum to 1.


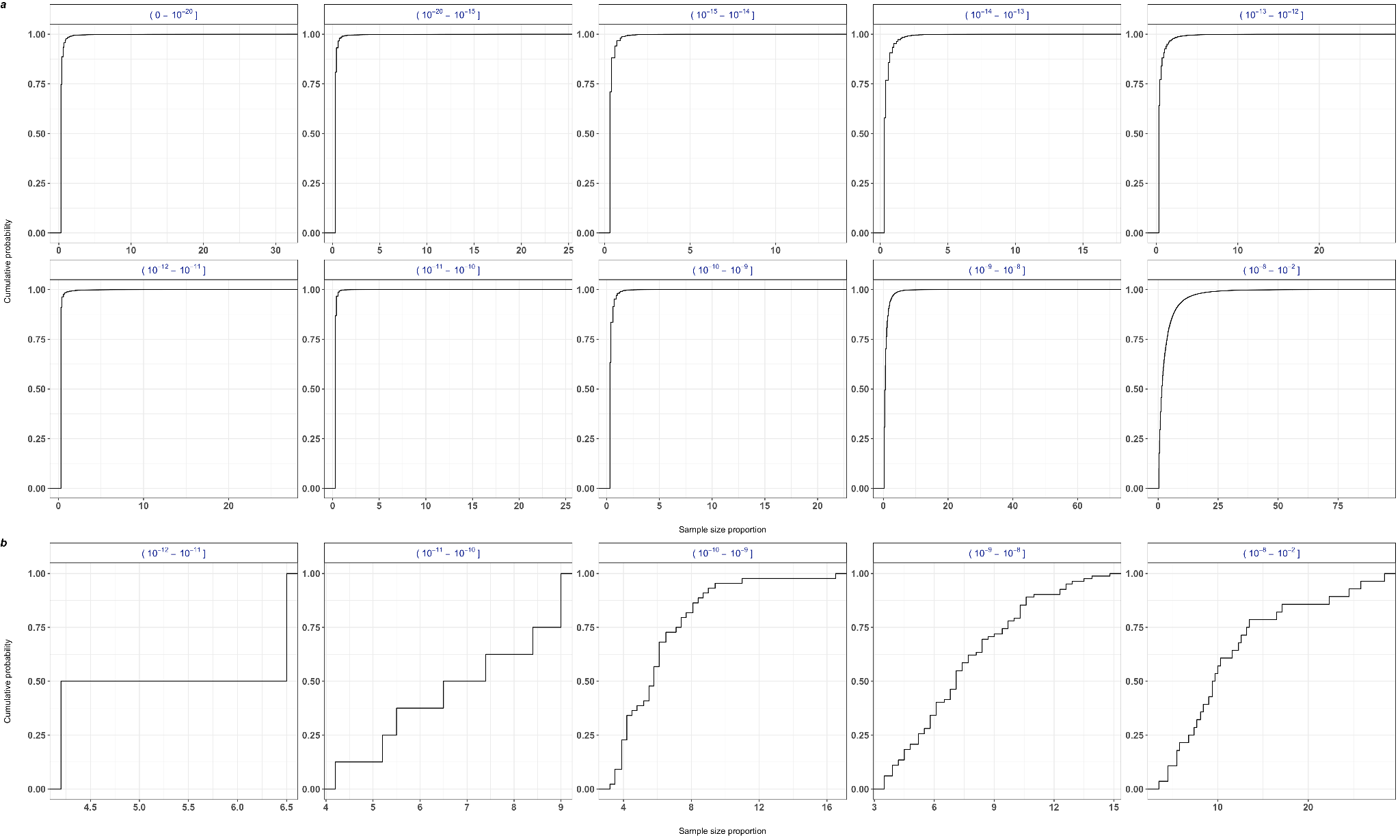


**Figure S5**: Empirical cumulative distribution of population incidence (sample size percentages on the x-axis) for different generation probability bins (sub-panels) for presumed signal sequences (***b***) and all the remaining public sequences (***a***) in a previously published large cohort study of TCR repertoires [[1]](https://www.zotero.org/google-docs/?83slJm).


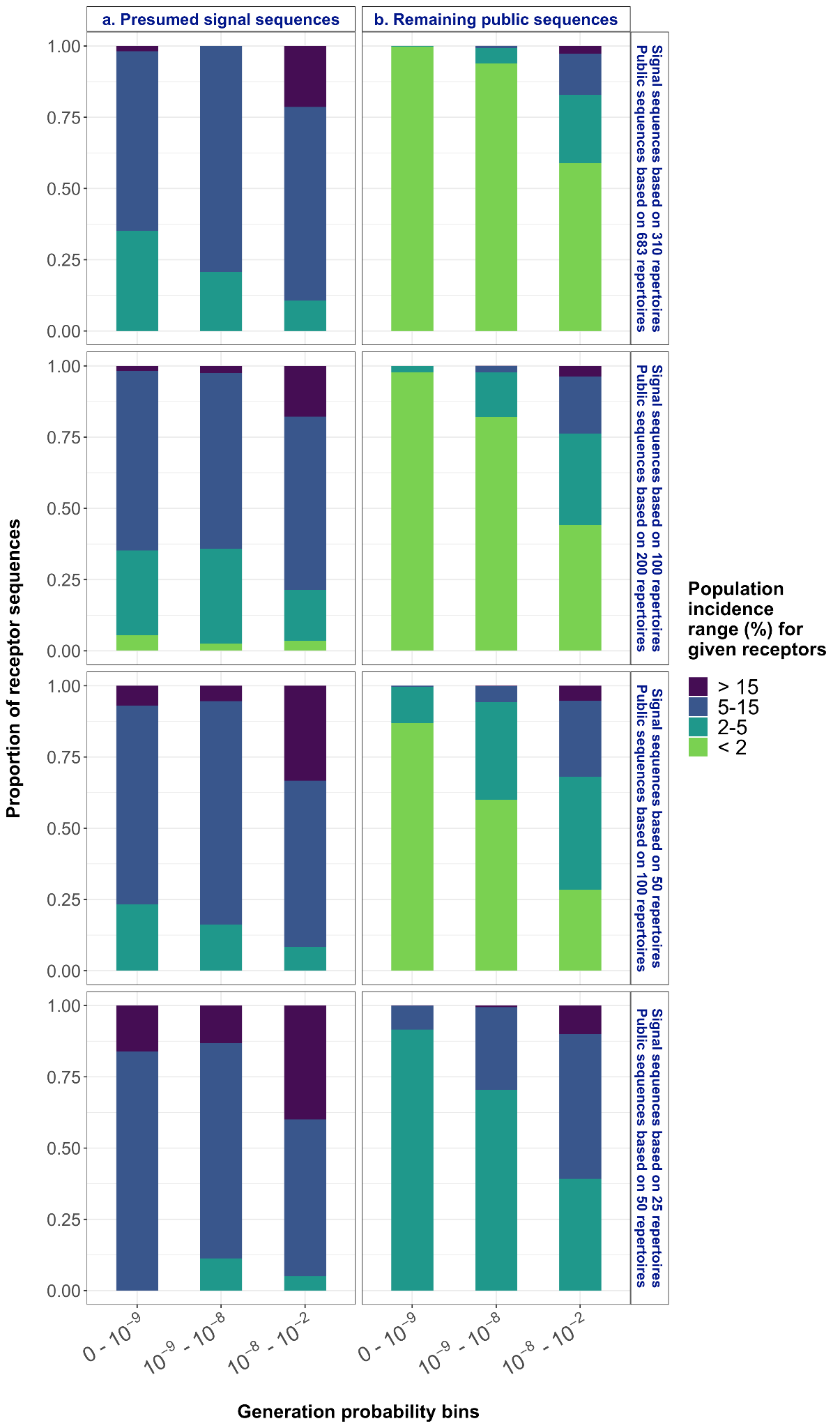


**Figure S6: Empirical distribution of population incidence for receptors within different generation probability intervals at different sample sizes (rows of the chart).** The relation between generation probability and population incidence of public AIR sequences was determined based on a previously published large cohort study of TCR repertoires [[1]](https://www.zotero.org/google-docs/?BD0ZDa) separately for presumed signal sequences (***a***) and all the remaining public sequences (***b***) at different sample sizes (rows of the chart)**.** In both ***a*** and ***b*,** receptors are along the x-axis split into three distinct bins according to their generation probability. The stacked bars along the y-axis represent the proportion of receptors within a given generation probability interval that have population incidence falling within a particular range (with a distinct colour representing each population incidence range). A full sample size of n=683 repertoires with approximately balanced labels (same chart as Figure 3) was shown for comparison with other investigated sample sizes of n = [200, 100, 50 repertoires] with balanced labels. Note that the bins of both generation probability and sample size proportions are half-open intervals that include the left endpoint but exclude the right endpoint. If the distribution of sequences at different population incidence levels (colours) within each generation probability interval (on the x-axis) at the full sample size of 683 repertoires (top row in the chart) is assumed as a proxy for the true population estimates, we notice that the true population estimates are well approximated with an increase in sample size (rows 2-4 in the chart).

**References**

[1. Emerson RO, DeWitt WS, Vignali M, Gravley J, Hu JK, Osborne EJ, et al.. Immunosequencing identifies signatures of cytomegalovirus exposure history and HLA-mediated effects on the T cell repertoire. *Nat Genet*. 2017; doi: 10.1038/ng.3822.](https://www.zotero.org/google-docs/?5a9o6R)

[2. Sacco K, Castagnoli R, Vakkilainen S, Liu C, Delmonte OM, Oguz C, et al.. Immunopathological signatures in multisystem inflammatory syndrome in children and pediatric COVID-19. *Nat Med*. Nature Publishing Group; 2022; doi: 10.1038/s41591-022-01724-3.](https://www.zotero.org/google-docs/?5a9o6R)
