# Supplementary material for: simAIRR: simulation of adaptive immune repertoires with realistic receptor sequence sharing for benchmarking of immune state prediction methods
Source: Gigascience. 2023 Oct 17;12:giad074. doi: 10.1093/gigascience/giad074 (PMC10580376; doi:10.1093/gigascience/giad074)
Supplement: giad074_Response_to_Reviewer_Comments_Original_Submission [file giad074_response_to_reviewer_comments_original_submission.pdf]

# Author's Response To Reviewer Comments

Uploaded the following two documents with responses:
- A cover letter to the editor
- A point by point response to the reviewers
