# Supplementary material for: simAIRR: simulation of adaptive immune repertoires with realistic receptor sequence sharing for benchmarking of immune state prediction methods
Source: Gigascience. 2023 Oct 17;12:giad074. doi: 10.1093/gigascience/giad074 (PMC10580376; doi:10.1093/gigascience/giad074)
Supplement: giad074_Response_to_Reviewer_Comments_Revision_1 [file giad074_response_to_reviewer_comments_revision_1.pdf]

Author's Response To Reviewer Comments

1) Please address the very minor follow-up request of reviewer #1 (see below).

Reviewer #1: The authors have addressed all reviewer comments and the manuscript is much clearer as a result. I have only one additional suggestion, which is to include indications in Figure 4 of previous model performances that the authors mention in the text as their comparison points.

Response: This minor suggestion is very good. However, the model performances from the previous studies do not include bounds for uncertainty estimates as in Figure 4 of this manuscript. Our intention in mentioning the model performances of previous studies was to talk about the trend of decreasing performance with either a decreasing sample size or witness rate, which is visible across the sub-panels of the figure (increasing from left to right). Including single numbers from previous studies could have worked well if those metrics were some form of average performance across multiple repetitions of the same setting. However, in their current form, those indications in isolation could be misleading. To avoid any misinterpretations, we instead added sentences in the Figure caption redirecting the readers to the relevant findings of the original studies themselves.

2) Please include a citation to your upcoming GigaDB dataset (including the DOI link) to your reference list, and cite this by number from the data availability section.

The citation is:

[xx] Kanduri C, Scheffer L, Pavlović M, Rand KD, Chernigovskaya M et al. Supporting data for "simAIRR: simulation of adaptive immune repertoires with realistic receptor sequence sharing for benchmarking of immune state prediction methods" GigaScience Database. 2023 http://dx.doi.org/10.5524/102434

In the data availability section, please write something along the lines, "An archival copy of the code and supporting data is available via the GigaScience repository, GigaDB [xx]".

Response: The suggested citation and description have been included.

3) Analogous to the example above, please also move all other online data sources (Zenodo, Github etc) to the bibliography (including the URL or DOI - DOI is preferred if available) and cite them by reference number from the text, rather than inserting the DOI/URL directly.

Response: All other online data sources (except the URL in the abstract) have been moved to the bibliography and cited by reference number from the text.

4) Please register your software in the bio.tools and SciCrunch.org databases to receive RRID (Research Resource Identification Initiative ID) and biotoolsID identifiers, and include these in your manuscript (in the "availability and requirements" section, below the "licence " line). This will facilitate tracking, reproducibility and re-use of your tool.

Response: RRID and biotoolsID have now been included in the manuscript under “availability and requirements”.

5) Please add ORCIDs for all authors to the title page, if available (we have these on our files:
ORCID iDs: Chakravarthi Kanduri [0000-0002-4783-9060]; Lonneke Scheffer [0000-0001-8900-075X]; Milena Pavlović [0000-0002-2484-3868]; Knut Dagestad Rand [0000-0003-3359-7619]; Maria Chernigovskaya [0000-0002-1507-4171]; Oz Pirvandy [0000-0001-7213-2043]; Gur Yaari [0000-0001-9311-9884]; Victor Greiff [0000-0003-2622-5032]; Geir K Sandve [0000-0002-4959-1409];

6) At this stage, you may remove tracking of changes/highlighting that was made for the purpose of peer review.

Response: Tracking has been removed now.

6) Please submit editable source files (e.g. .doc, .docx, LaTex...) for the text (we only have a pdf so far)

Response: A .docx file has now been included.
