# Supplementary material for: simAIRR: simulation of adaptive immune repertoires with realistic receptor sequence sharing for benchmarking of immune state prediction methods
Source: Gigascience. 2023 Oct 17;12:giad074. doi: 10.1093/gigascience/giad074 (PMC10580376; doi:10.1093/gigascience/giad074)
Supplement: giad074_Reviewer_1_Report_Original_Submission — Chaim Schramm -- 4/4/2023 Reviewed [file giad074_reviewer_1_report_original_submission.pdf]

Reviewer Report

Title: simAIRR: simulation of adaptive immune repertoires with realistic receptor sequence sharing for benchmarking of immune state prediction methods

Version: Original Submission **Date:** 4/4/2023

Reviewer name: Chaim Schramm

Reviewer Comments to Author:

This manuscript by Kanduri et al describes "simAIRR," a new framework for simulating B and T cell receptor repertoires. The goal is to more accurately capture the long tail of "outlier" rearrangements in naturally occurring repertoires. The authors thus aim to provide better training data for machine learning models to avoid "shortcut learning." This is a novel advance over previous simulators and will help the nascent application of machine learning to immunology continue to grow.Major concerns:1. My biggest concern is the interpretability of Fig 3. Despite reading the caption multiple times, I am still not entirely sure I understand what is being shown. Perhaps this is partly because the bins chosen seem unintuitive. The patterns of values are also surprising, such as 0.27 in the min x=[1.9,5.1), y=[8,2). Also the fact that the distribution is concentrated in x=[02,0.4) for y=[11,10) and [12,11) but the spread out again for y=[13,12) and [14,13). Is there perhaps a way to show these data as figures (perhaps multiple) rather than tables?2. The authors emphasize throughout the paper that their goal is to enable study of all AIRR loci, but then acknowledge at the end that users will need to gather their own training data for anything other than TRB. Would it be possible for the authors to at least offer more guidelines for how much and what type of data is needed? Are there scripts/tutorials available for how to go about this, or details on what the output file should look like?3. In describing Fig 4, the authors seems to be saying that machine learning models perform equally well on data generated by simAIRR as on data from previous simulators. I assume this is not what they intend, and so should at least be clarified. It might also be useful to do a direct comparison of the same ML model on different simulation data to reinforce what is shown in Fig 2 and fully demonstrate the advantages of simAIRR.Minor remarks:1. The equation on page 15 is displayed only as empty boxes in my PDF.

Level of Interest

Please indicate how interesting you found the manuscript: Choose an item.

Quality of Written English

Please indicate the quality of language in the manuscript: Choose an item.

Declaration of Competing Interests

Please complete a declaration of competing interests, considering the following questions:

- Have you in the past five years received reimbursements, fees, funding, or salary from an organisation that may in any way gain or lose financially from the publication of this manuscript, either now or in the future?
- Do you hold any stocks or shares in an organisation that may in any way gain or lose financially from the publication of this manuscript, either now or in the future?
- Do you hold or are you currently applying for any patents relating to the content of the manuscript?
- Have you received reimbursements, fees, funding, or salary from an organization that holds or has applied for patents relating to the content of the manuscript?
- Do you have any other financial competing interests?
- Do you have any non-financial competing interests in relation to this paper?

If you can answer no to all of the above, write 'I declare that I have no competing interests' below. If your reply is yes to any, please give details below.

I declare that I have no competing interests.

I agree to the open peer review policy of the journal. I understand that my name will be included on my report to the authors and, if the manuscript is accepted for publication, my named report including any attachments I upload will be posted on the website along with the authors' responses. I agree for my report to be made available under an Open Access Creative Commons CC-BY license (http://creativecommons.org/licenses/by/4.0/). I understand that any comments which I do not wish to be included in my named report can be included as confidential comments to the editors, which will not be published.

Choose an item.

To further support our reviewers, we have joined with Publons, where you can gain additional credit to further highlight your hard work (see: https://publons.com/journal/530/gigascience). On publication of this paper, your review will be automatically added to Publons, you can then choose whether or not to claim your Publons credit. I understand this statement.

Yes Choose an item.
