# Supplementary material for: simAIRR: simulation of adaptive immune repertoires with realistic receptor sequence sharing for benchmarking of immune state prediction methods
Source: Gigascience. 2023 Oct 17;12:giad074. doi: 10.1093/gigascience/giad074 (PMC10580376; doi:10.1093/gigascience/giad074)
Supplement: giad074_Reviewer_2_Report_Original_Submission — William Lees -- 4/25/2023 Reviewed [file giad074_reviewer_2_report_original_submission.pdf]

Reviewer Report

Title: simAIRR: simulation of adaptive immune repertoires with realistic receptor sequence sharing for benchmarking of immune state prediction methods

Version: Original Submission **Date:** 4/25/2023

Reviewer name: William Lees

Reviewer Comments to Author:

Kanduri et al. describe a novel and important approach to enhance the realism of simulated adaptive immune repertoires and hence reduce the ability of learning models to take unintended shortcuts. Improvements such as this are badly needed, to provide realistic training sets to the field.While the manuscript overall is well structured and clearly written, I found it hard to follow the description of the approach. It is described in various places in terms of the 'empirical relation between generation probability and population incidence'. The probability distribution shown in the tables of Fig. 3 is of course an empirically determined relationship, but the references to it distract from the overall message, particularly in the earlier sections. For example, in the Abstract, "simAIRR constructs antigen-experienced-like baseline repertoires by introducing signals that follow the empirical relationship between VDJ generation probability and population incidence of public sequences calibrated on real-world experimental datasets". The sentence could be simplified to state that simAIRR constructs repertoires that more truly follow the observed population distribution, compared to repertoires constructed solely on the basis of generation probabilities. Likewise on page 3, "By deriving an empirical relation between the population incidence and generation probability of sequences, it may be possible to identify sequences that are observed with unlikely high population incidence given their generation probability". It would be simpler to say "By comparing the population incidence with the generation probability…". Perhaps the authors could also consider a shorter term than the 8-word phrase, so that once the form of the tables is introduced in Fig, 3, it can be referred to more succinctly. Finally, it would be helpful to restructure the description of the simAIRR approach on page 7 so that the overall mechanism is described first, rather than postponing it to the beginning of the second paragraph.In Figure 3, it would be clearer to express probability ranges as numbers (e.g 10-Â² - 10-â¸ on the first row) rather than using negative logs. "The row-sums of the heatmaps should sum to 1" - not sure this is necessary text, but, if included, please check that they do, and report as such.Case study 1: "As expected, we observed that the performance of the suited ML method was close to perfect when the witness rate was equal to or above 10 sequences per 10âµ sequences" - please explain why this is "as expected".Discussion - In a real-world repertoire, outlier sequences may be correlated, i.e., the likelihood of finding one in a repertoire may depend on the presence or absence of another, where both sequences are associated with the same immune stimulus. Similarly, there may be correlation between outliers and 'signal' sequences, where, for example, a particular antigen experienced in the past primes for a specific response to the condition being investigated. These points may be worth mentioning in the discussion.The manuscript addresses AIRRs in general, but the application of simAIRR to BCR repertoires is not discussed. Could the authors please clarify whether simAIRR is intended for use with BCRs as well

as TCRs? BCRs would seem to raise some additional challenges posed by the presence of clonal lineages, and it would be useful to have some thoughts in the discussion on these, one way or the other.simAIRR itself is well documented, with several detailed tutorials covering particular use cases. It is helpfully provided both as a Python package and a ready-to-run container. Could the authors please include example code to create the public- and signal- pgen_count_mapping files?

Level of Interest

Please indicate how interesting you found the manuscript: Choose an item.

Quality of Written English

Please indicate the quality of language in the manuscript: Choose an item.

Declaration of Competing Interests

Please complete a declaration of competing interests, considering the following questions:

- Have you in the past five years received reimbursements, fees, funding, or salary from an organisation that may in any way gain or lose financially from the publication of this manuscript, either now or in the future?
- Do you hold any stocks or shares in an organisation that may in any way gain or lose financially from the publication of this manuscript, either now or in the future?
- Do you hold or are you currently applying for any patents relating to the content of the manuscript?
- Have you received reimbursements, fees, funding, or salary from an organization that holds or has applied for patents relating to the content of the manuscript?
- Do you have any other financial competing interests?
- Do you have any non-financial competing interests in relation to this paper?

If you can answer no to all of the above, write 'I declare that I have no competing interests' below. If your reply is yes to any, please give details below.

I declare that I have no competing interests

I agree to the open peer review policy of the journal. I understand that my name will be included on my report to the authors and, if the manuscript is accepted for publication, my named report including any attachments I upload will be posted on the website along with the authors' responses. I agree for my report to be made available under an Open Access Creative Commons CC-BY license (http://creativecommons.org/licenses/by/4.0/). I understand that any comments which I do not wish to be included in my named report can be included as confidential comments to the editors, which will not be published.

Choose an item.

To further support our reviewers, we have joined with Publons, where you can gain additional credit to further highlight your hard work (see: https://publons.com/journal/530/gigascience). On publication of this paper, your review will be automatically added to Publons, you can then choose whether or not to claim your Publons credit. I understand this statement.

Yes Choose an item.
