# Supplementary material for: simAIRR: simulation of adaptive immune repertoires with realistic receptor sequence sharing for benchmarking of immune state prediction methods
Source: Gigascience. 2023 Oct 17;12:giad074. doi: 10.1093/gigascience/giad074 (PMC10580376; doi:10.1093/gigascience/giad074)
Supplement: giad074_Reviewer_3_Report_Original_Submission — Robert Sinkovits -- 5/1/2023 Reviewed [file giad074_reviewer_3_report_original_submission.pdf]

Reviewer Report

Title: simAIRR: simulation of adaptive immune repertoires with realistic receptor sequence sharing for benchmarking of immune state prediction methods

Version: Original Submission **Date:** 5/1/2023

Reviewer name: Robert Sinkovits

Reviewer Comments to Author:

The authors present a novel and valuable method for generating synthetic adaptive immune receptor repertoires (AIRR) that properly account for immune state. While other methods already exist for repertoire generation, they rely purely on statistical models of VDJ recombination, with the consequence that any overlap between individuals - referred to as public sequences - is due to convergent recombination rather than shared exposure to the same antigen. The observed overlap in repertoires can be dominated by sequences that are "easy" to generate, for example, those that involve heavily used genes and minimal insertions or deletions at the VD and DJ junctions. These more realistic repertoires can be valuable when evaluating the performance of machine learning (ML) models in clinical and research settings. The authors put significant effort into making the software usable, including the creation of a GitHub repo, Python package and Docker container.While I'm supportive of this work, my biggest concern is that the testing was restricted to sequences from T cell receptor beta chains (TCRB). It's well known that a large fraction of the T cells in CMV (cytomegalovirus) positive individuals are specific to CMV, with estimates ranging from 30% to as high as 90%. Fortunately, I believe that the experimental data set that was used (Emerson et al., Nature Genetics) distinguishes between CMV positive and negative individuals. I suggest repeating the testing with the CMV negative cohort to confirm that the results are still valid. I would also like to see testing extended to B cell heavy chains. Unlike T cells, B cells undergo somatic hypermutation (SHM) which adds significant diversity and complexity to the repertoire. It could be valuable to demonstrate that the new approach works as expected for B cells. Otherwise, I might recommend that the title be changed to "simulation of adaptive T cell repertoires …"I have a few other minor comments.If possible, please try to reduce the length of the figure captions, especially figure 1. While long captions are often unavoidable in the biomedical literature due to the complexity of the figures, try to at least eliminate some of the redundant content that is covered in the main text.This might be a limit in my comprehension or just an issue of semantics, but I'm a little confused by the way the term "outlier" is used, particularly as shown in figure 1. I would expect the public sequences generated by convergent recombination to not be considered outliers because they will occur at the frequency predicted by the statistical models. Only the sequences that are selected by exposure to antigen will deviate from the predictions and be considered outliers.

Level of Interest

Please indicate how interesting you found the manuscript: Choose an item.

Quality of Written English

Please indicate the quality of language in the manuscript: Choose an item.

Declaration of Competing Interests

Please complete a declaration of competing interests, considering the following questions:

- Have you in the past five years received reimbursements, fees, funding, or salary from an organisation that may in any way gain or lose financially from the publication of this manuscript, either now or in the future?
- Do you hold any stocks or shares in an organisation that may in any way gain or lose financially from the publication of this manuscript, either now or in the future?
- Do you hold or are you currently applying for any patents relating to the content of the manuscript?
- Have you received reimbursements, fees, funding, or salary from an organization that holds or has applied for patents relating to the content of the manuscript?
- Do you have any other financial competing interests?
- Do you have any non-financial competing interests in relation to this paper?

If you can answer no to all of the above, write 'I declare that I have no competing interests' below. If your reply is yes to any, please give details below.

I declare that I have no competing interests

I agree to the open peer review policy of the journal. I understand that my name will be included on my report to the authors and, if the manuscript is accepted for publication, my named report including any attachments I upload will be posted on the website along with the authors' responses. I agree for my report to be made available under an Open Access Creative Commons CC-BY license (http://creativecommons.org/licenses/by/4.0/). I understand that any comments which I do not wish to be included in my named report can be included as confidential comments to the editors, which will not be published.

Choose an item.

To further support our reviewers, we have joined with Publons, where you can gain additional credit to further highlight your hard work (see: https://publons.com/journal/530/gigascience). On publication of this paper, your review will be automatically added to Publons, you can then choose whether or not to claim your Publons credit. I understand this statement.

Yes Choose an item.
